# Bone Radiopharmaceuticals: Evolution from Palliative Therapy to Targeted Precision Oncology and Theranostics

**DOI:** 10.3390/biology15191784

**Published:** 2026-10-08

**Authors:** Andras Polyak, Christiane Matuschek

**Affiliations:** 1 Radiation Therapy and Radiation Oncology, Medical School OWL (East Westphalia-Lippe), Bielefeld University, Morgenbreede 1, D-33615 Bielefeld, Germany; 2 Department of Radiation Oncology, Klinikum Bielefeld Mitte, Medical School and University Medical Center East Westphalia-Lippe, Bielefeld University, Teutoburger Str 50, D-33604 Bielefeld, Germany

**Keywords:** bone radiopharmaceuticals, bone tumor, bone metastases, skeletal scintigraphy, theranostics, bisphosphonates, alpha emitters, Auger electrons

## Abstract

Bone radiopharmaceuticals have evolved from early palliative agents into integral components of targeted precision oncology and theranostics. This review comprehensively traces the historical trajectory, pathophysiological mechanisms, and clinical applications of bone-directed nuclear medicine. We contrast primary bone malignancies—which directly produce malignant osteoid matrix—with metastatic bone disease, which colonizes the endosteal niche via the RANK/RANKL/OPG axis and the metastatic vicious cycle. The molecular mechanisms governing skeletal radioisotope localization are systematically detailed, encompassing calcium mimetics (^223^Ra), inorganic phosphate integration (^32^P), hydroxyapatite chemisorption (^99m^Tc-labeled diphosphonates), isomorphous surface exchange ([^18^F]NaF), and direct biomarker targeting. Furthermore, we highlight the therapeutic transition from medium-range beta-emitters to high-linear energy transfer (LET) targeted alpha therapy (^225^Ac), nanometer-scale Auger electron emitters, and cell-directed theranostic vectors. By unifying radiopharmaceutical chemistry, microdosimetry, and emerging artificial intelligence-driven personalized dosimetry, this review provides a strategic roadmap for advancing precision bone endoradiotherapy.

## Simple Summary

Bone radiopharmaceuticals have advanced from early palliative treatments for bone metastases to highly specific precision oncology and theranostics. This review outlines the historical development of skeletal nuclear medicine, comparing the pathophysiological differences between primary bone malignancies and metastatic bone disease. It details how bone-seeking radioisotopes interact with the skeleton—from early calcium mimetics and technetium-labeled bisphosphonates to fluorine-18 PET imaging. Furthermore, it highlights the paradigm shift from indirect bone remodeling markers toward direct cell- and microenvironment-targeted agents, including targeted alpha therapy, Auger electron emitters, and theranostic pairs like FAPI and PSMA ligands. Finally, the article discusses future directions, including artificial intelligence-driven dosimetry and novel combination therapies for personalized patient care.

## 1. Introduction

The human skeleton is a highly sophisticated, metabolically active organ, yet it represents the most frequent site of distant metastasis for many solid tumors, most notably breast and prostate carcinomas [1]. Statistically, advanced stages of these malignancies demonstrate an overwhelming tropism for the bone matrix, with autopsy studies revealing skeletal involvement in up to 70% to 80% of patients who succumb to breast or prostate cancer. Given this high prevalence, vigilant screening for skeletal involvement, precise monitoring, and prompt therapeutic interventions have become foundational pillars of modern oncological care.

When disseminated tumor cells infiltrate the bone microenvironment, they disrupt physiological bone turnover, leading to severe skeletal complications. This disruption triggers a destructive cascade that manifests clinically as severe bone pain, pathological fractures, and life-threatening neurological or systemic complications. These clinical milestones are collectively categorized as skeletal-related events (SREs), which primarily encompass pathological fractures, spinal cord compression, the necessity for bone surgery or radiation therapy, and tumor-induced hypercalcemia. SREs inflict a substantial clinical burden on patient quality of life and are remarkably common in the metastatic population, occurring on average every 3 to 6 months if left unchecked [1].

At the microscopic level, the skeletal system maintains its structural integrity through a continuous, finely tuned remodeling process governed by the coupled actions of bone-forming osteoblasts and bone-resorbing osteoclasts. This homeostatic cycle not only ensures mechanical strength but also serves as a central hub for systemic calcium and phosphate regulation. Malignant cells exploit these very pathways, hijacking the local cellular machinery to create either predominantly osteoblastic (bone-building) or osteolytic (bone-destroying) lesions.

Nuclear medicine stands uniquely equipped to counter this clinical challenge by utilizing physiological skeletal turnover for targeted imaging and radionuclide therapy. Bone radiopharmaceuticals are engineered to exploit these natural, tumor-altered biochemical processes. Over the decades, the field has transitioned from utilizing rudimentary calcium mimetics, simple ion-exchange mechanisms, and surface chemisorption—which defined the early eras of conventional SPECT imaging and primitive palliative therapy—toward designing highly sophisticated, target-specific molecular vectors. Modern nuclear oncology increasingly relies on advanced receptor-ligand interactions and metabolic pathways to deliver precise, high-affinity PET (positron emission tomography) and SPECT (single-photon emission computed tomography) agents.

### 1.1. Primary Bone Malignancies vs. Metastatic Bone Disease: Pathophysiological and Radiopharmaceutical Considerations

While clinical endpoints (e.g., survival benefits and adverse event rates) represent the ultimate measures of clinical success, the primary focus of this review centers on the molecular mechanisms, radiochemical design, and microdosimetric radiobiology of the radiopharmaceuticals used for bone malignancies and metastatic bone tumors.

A fundamental distinction in nuclear bone imaging and targeted radionuclide therapy (TRT) lies in the underlying pathophysiology of primary bone tumors compared to metastatic bone lesions. Primary bone malignancies, such as osteosarcoma, Ewing sarcoma, and chondrosarcoma, originate directly from mesenchymal bone or cartilage progenitor cells but present distinct histological features and uptake mechanisms across radiopharmaceutical classes. Osteosarcoma is characterized by direct production of malignant osteoid matrix, leading to intense uptake of conventional bone-seeking agents via localized neo-ossification, mineral exchange, and osteoblastic activity. In contrast, Ewing sarcoma is an aggressive small round-cell tumor devoid of osteoid matrix that predominantly causes osteolytic bone destruction; uptake of conventional bone-seeking tracers in Ewing sarcoma is mainly driven by regional hyperemia and non-specific peritumoral reactive osteogenesis rather than direct tumor matrix mineralization. Consequently, direct metabolic probes such as [^18^F]FDG provide vastly superior lesion depiction and clinical utility in Ewing sarcoma. Chondrosarcoma, producing cartilaginous matrix, demonstrates variable uptake depending on histological grade and calcification density. Thus, radiopharmaceutical accumulation in primary bone tumors exhibits substantial heterogeneity governed by underlying tumor histology [2,3,4,5].

Conversely, bone metastases, predominantly originating from carcinomas of the prostate, breast, lung, and kidney, represent secondary colonization of the endosteal niche. Metastatic tumor cells rarely produce structural bone matrix themselves; instead, they hijack the host bone microenvironment, triggering either a reactive host osteoblastic response (sclerotic metastases) or hyperactive osteoclastic destruction (lytic metastases).

Crucially, this pathophysiological dichotomy dictates the mechanism of radiopharmaceutical uptake, establishing two distinct functional classes:

#### 1.1.1. Bone-Matrix-Targeting (Conventional) Agents

Standard bone-seeking radiopharmaceuticals (e.g., ^99m^Tc-bisphosphonates, [^18^F]NaF, and ^223^Ra) do not directly bind neoplastic cells. Instead, they localize to areas of host-reactive mineral exchange and osteoblastic turnover. While effective for sclerotic remodeling, they exhibit inherent diagnostic and therapeutic blind spots in purely osteolytic lesions or early intramedullary marrow colonization where reactive bone formation is absent [4,5].

#### 1.1.2. Direct Tumor- and Microenvironment-Targeting (Non-Bone-Seeking) Agents

To overcome matrix-dependent limitations, modern theranostics utilizes vectors originally developed for non-osseous targets, such as prostate-specific membrane antigen (PSMA) ligands, fibroblast activation protein inhibitors, and somatostatin receptor analogues. These radiopharmaceuticals target cell-surface antigens, altered metabolism, or cancer-associated fibroblasts (CAFs) directly. By binding to neoplastic cells or their supporting stroma rather than the mineralized matrix, these direct probes identify osteolytic and marrow metastases independently of host bone remodeling, bridging the gap between skeletal and extra-skeletal disease [6,7,8,9].

### 1.2. Search Strategy and Literature Selection

To compile the literature for this narrative review, a comprehensive search was conducted across PubMed/MEDLINE, Web of Science, and Scopus databases up to August 2026. Priority was given to seminal historical publications establishing fundamental, authentic physical, pharmacological principles, high-impact research findings, systematic reviews, and recent primary literature (2020–2026) reflecting state-of-the-art precision oncology advancements. Articles not indexed in English or lacking peer review were excluded.

## 2. The Beginnings and the Palliative Era (1930s–1960s)

Although radiopharmaceuticals are primarily known to the public and in modern clinical practice as high-resolution molecular tools for diagnostic nuclear medicine, their historical trajectory did not originate from the intentional design of targeted radiotracers. Instead, the field was catalyzed by unfortunate workplace accidents and the subsequent realization of their palliative medical applications. Over the decades, systemic radionuclide therapy for bone metastases has undergone multiple technological iterations, beginning initially with non-tumor-specific, bone-seeking elements that relied entirely on the natural physiological pathways of the skeleton.

This relationship between radiation and skeletal physiology was first recognized through occupational exposure in watch dial painters. The innate capacity of certain radionuclides to target and accumulate within the skeletal system was initially uncovered through clinical observations of workers employed as luminous watch dial painters. These individuals routinely licked their brushes to maintain a fine point, resulting in the chronic ingestion of radium. Over time, this chronic exposure caused them to suffer from severe bone necrosis, debilitating osteomyelitis, and malignant bone tumors [10,11]. While the scientific community first attributed this phenomenon to non-specific tracer entrapment within the reticuloendothelial cells of the bone marrow, subsequent post-mortem autoradiographic analyses of human tissue conclusively refuted this theory. Instead, these investigations demonstrated that the radioactivity was chemically locked directly within the mineralized bone structure itself [12].

While these occupational accidents revealed the skeletal deposition of heavy radioactive elements, the broader genesis of systemic skeletal radionuclide therapy was deeply intertwined with the foundational breakthroughs of 20th-century nuclear physics and the conceptualization of the tracer principle. The groundwork for using isotopes to track biological systems was laid by George de Hevesy and Fritz Paneth in their landmark 1913 study, “The Solubility of Lead Sulphide and Lead Chromate.” This research was the first to utilize radioisotopes as tracers—specifically employing labeled lead in the form of Thorium B or Radium D—to precisely measure the solubility of highly insoluble chemical compounds. This pioneering work fundamentally demonstrated the absolute chemical identity of radioisotopes and their stable counterparts [13]. Building upon this realization, Hevesy’s subsequent pioneering experiments with radioactive lead in fava beans firmly established that radioisotopes behave identically both chemically and biologically to stable elements. This crucial milestone allowed them to function as highly reliable indicators for mapping complex, otherwise invisible physiological processes.

Expanding his vision from heavy metals to life-sustaining elements, Hevesy became the first investigator to introduce Phosphorus-32 (^32^P) into biological research. For generations, the scientific community had long maintained that bone was a metabolically stagnant and inert tissue—a concept that persisted until experimental models successfully proved that [^32^P]phosphate actively integrates into the skeletal matrix of mature rats [14]. These animal experiments showed clearly that bone metabolism was a highly dynamic, ongoing process rather than a static state. As noted in the original findings: “*From our experiments, it follows that the average time which a phosphorus atom thus spends in the organism of a normally fed rat is about two months.*” By systematically following the distribution of ^32^P across various organs and the skeleton, researchers were led to the revolutionary conclusion that “*… the formation of bones is a dynamic process, the bone continuously taking up phosphorus atoms which are partly or wholly lost again, and are replaced by other phosphorus atoms. In the case of an adult rat, about 30% of the phosphorus atoms deposited in the skeleton were removed in the course of twenty days.*” [14].

Hevesy’s fundamental realization of dynamic skeletal turnover, however, required a reliable supply of artificial isotopes to be viable on a larger scale. Fortunately, Ernest Lawrence’s development of the cyclotron in the 1930s, coupled with the subsequent discovery of artificial radioactivity, provided the necessary technical infrastructure for broad medical applications. In his initial ^32^P research, Hevesy had to meticulously prepare the isotope by bombarding sulfur (in the form of CS_2_) with neutrons from a rudimentary source, such as radon-beryllium. Because the yields of ^32^P produced in this inefficient manner were quite low, Hevesy contacted E. O. Lawrence directly at the University of California, Berkeley, requesting cyclotron-produced samples of ^32^P possessing significantly higher specific activity [15].

With cyclotron production rendering artificial radionuclides more accessible, medicine entered a new phase of exploration. While early clinical efforts focused heavily on exploiting the thyroid-iodine relationship for endocrine disorders, investigators soon recognized the immense potential for bone-seeking radionuclides to address the profound morbidity associated with advanced bone metastases. This initial period, spanning from the late 1930s through the 1960s, is historically characterized as the “Palliative Era.” During this time, the primary clinical objective was strictly the mitigation of intractable bone pain and the improvement of patient quality of life, rather than the extension of overall survival [16,17].

**Figure 1:**
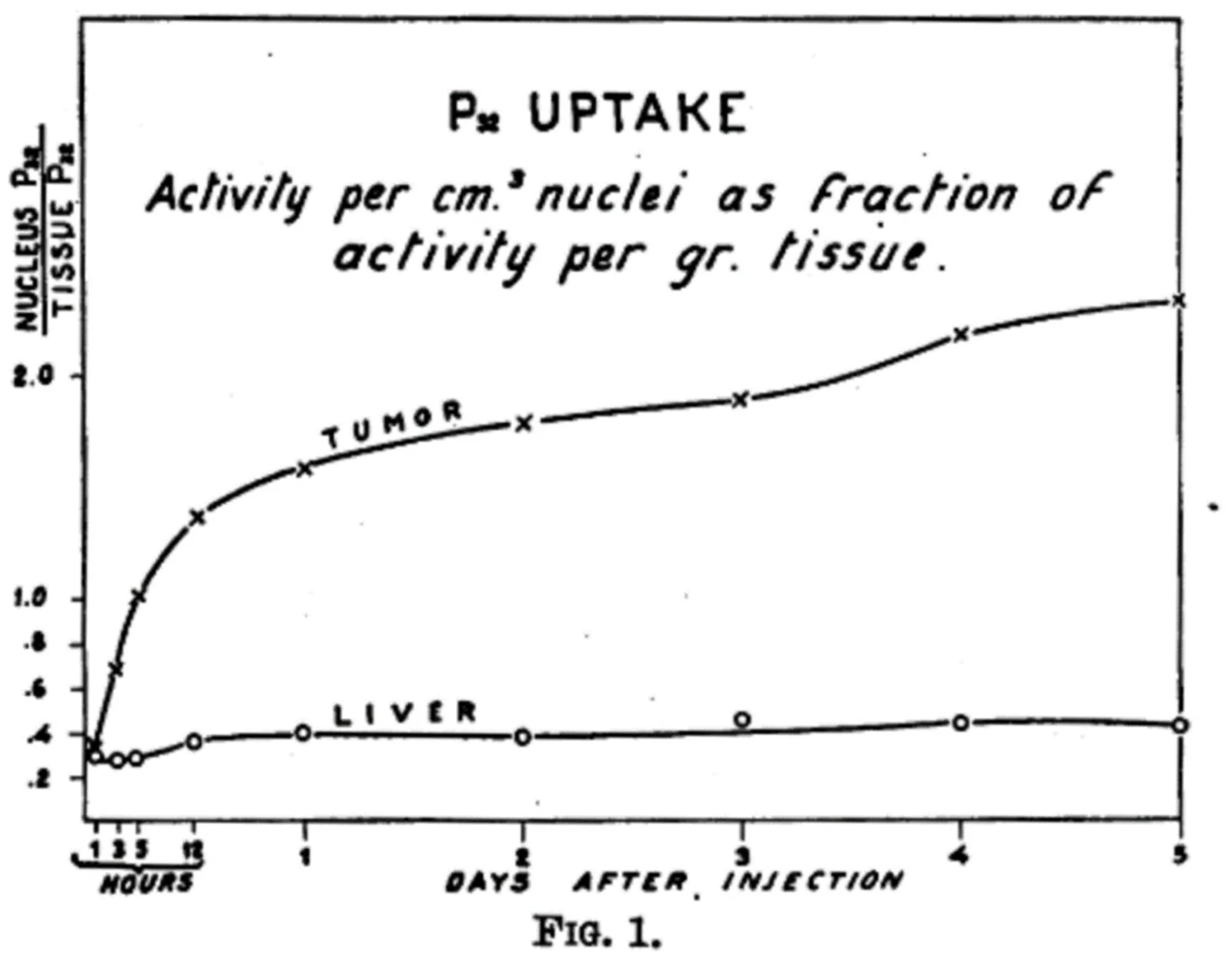
^32^P uptake in tumor and liver by Marshak et al. in 1940 [18].

The first true milestone in systemic bone therapy was achieved with the introduction of ^32^P administered in the form of an inorganic orthophosphate (Na_3_PO_4_, trisodium-phosphate, sodium-ortophosphate) [18,19,20] (Figure 1). During the late 1930s and early 1940s, researchers observed that intravenously administered [^32^P]orthophosphate accumulated significantly more in tumor-affected bone sites than in healthy tissue. This preferential localization was primarily driven by the pronounced reactive bone formation (osteoblastic response) and the accelerated mineral turnover surrounding osseous metastases [21]. Recognizing this therapeutic potential, Friedell and Storaasli published their landmark 1942 exploration into the therapeutic use of ^32^P specifically for the relief of severe bone pain in advanced breast cancer patients. This historic publication marked the true birth of unsealed source radionuclide therapy for skeletal lesions [21]. The underlying mechanism of action for ^32^P involves its direct incorporation into the hydroxyapatite mineral phase of the bone matrix. Structurally, [^32^P]orthophosphate exhibits a direct affinity for the bone, linking into the hydroxyapatite crystal lattice together with calcium and the hydroxyl moieties present in the skeletal bone mineral matrix. Wherever osteoblastic activity is pathologically increased due to tumor invasion, these therapeutic radiotracers readily react with the newly produced, hydrated amorphous calcium phosphate salts and emerging hydroxyapatite crystals [16,17]. Once fixed at the lesion site, the isotope delivers high-energy beta radiation directly to both the surrounding tumor cells and the immediate bone microenvironment.

Although pain response rates with [^32^P]orthophosphate were remarkably high—ranging from 50% to over 90% across various skeletal malignancies—its long-term clinical utility was severely hampered by its unfavorable physical properties. Emitting a maximum beta energy of 1.71 MeV with a substantial tissue penetration range of up to 8 mm, ^32^P inadvertently delivered a massive, non-targeted radiation dose to the nearby hematopoietic bone marrow. This crossfire effect resulted in significant, dose-dependent myelosuppression, often manifesting clinically as severe pancytopenia and, in rare instances, leading to the development of secondary acute leukemias [22,23,24].

As a consequence of these hematological toxicities, scientific interest shifted away from phosphorus and toward heavy alkaline earth metals, which are characterized by their natural calcimimetic behavior. Because elements such as strontium, barium, and radium belong to Group 2 of the periodic table, they naturally exist as divalent cations (M^2+^). Consequently, they follow the exact physiological pathways of calcium in the human body, incorporating directly into the inorganic bone matrix without requiring a complex chemical carrier [23,25].

Among these calcimimetic options, Strontium-89 (^89^Sr), administered as strontium chloride (commercially known as Metastron), emerged as a far more viable and safer alternative to [^32^P]orthophosphate. It offered a prolonged physical half-life of 50.5 days combined with a much shorter average beta tissue range of 2.4 mm, which provided a significantly more favorable therapeutic window by reducing bone marrow toxicity while fully maintaining analgesic efficacy. The earliest account of detailed clinical and postmortem studies with radioactive strontium in a group of patients with demonstrated metastatic carcinoma provided definitive proof of its targeting efficiency. Utilizing precise ash analysis and autoradiographic methods, Treadwell and colleagues elegantly showed a highly increased accumulation of ^89^Sr in both metabolically active normal bone and, to an even greater extent, across a variety of primary bone tumors [3].

In a parallel quest to optimize skeletal targeting during the late 1950s, investigators also utilized the pure beta emitters Calcium-45 (^45^Ca) and Calcium-47 (^47^Ca) to meticulously map human calcium metabolism and perform experimental palliative treatments for bone metastases [26,27,28,29]. While ^45^Ca was eventually deemed entirely unsuitable for routine clinical use due to its excessively long physical half-life of 162.6 days (which led to prolonged retained radiation), these early trials provided critical, foundational pharmacokinetic data that ultimately led to the clinical prioritization of ^89^Sr.

Concurrently, the 1950s and 1960s featured extensive tracking of calcium-mimetic pathways. Alongside investigations into complex chelates of rare earth elements and therapeutic lanthanides [30,31], several isotopes of the alkaline earth metal barium were thoroughly evaluated for their skeletal localization properties. By deploying complex animal models and human subjects, researchers compared the detailed biodistribution of Barium-140 (^140^Ba) and Barium-133 (^133^Ba) directly against calcium and strontium [25,32]. Ultimately, these comparative studies established that barium isotopes cleared from the bloodstream and accumulated within the bone matrix twice as fast as native calcium, mimicking the kinetic profiles of therapeutic radium isotopes.

Despite these profound thermodynamic and pharmacokinetic discoveries regarding bone-seeking isotopes during this era, diagnostic imaging remained a significant bottleneck. Without the hardware and imaging capabilities to safely scan and quantify the distribution of these therapeutic agents in vivo, clinicians were constrained by the lack of in vivo imaging technology, a challenge that would only be resolved with the subsequent dawn of modern diagnostic nuclear medicine instrumentation in the decades that followed.

## 3. The Lost and Found Fluorine-18

By the close of the 1960s, the “Palliative Era” had reached a critical crossroads. While first-generation therapeutic radionuclides like ^32^P and ^89^Sr could provide essential symptomatic relief to advanced cancer patients suffering from the devastating “vicious cycle” of malignant bone destruction and bone pain, they remained biologically non-specific tools that exacted a high toxicological cost on the bone marrow. The growing clinical realization that pure, non-targeted palliation was insufficient for comprehensive patient management necessitated a twofold paradigm shift: the chemical synthesis of more stable bone-seeking carrier molecules and the development of higher-resolution diagnostic isotopes. Consequently, early nuclear medicine clinicians often had to rely on crude metabolic response data and laboratory systemic markers rather than precise, high-fidelity anatomical visualization to guide their palliative therapies.

A highly promising candidate to bridge this diagnostic gap had actually emerged early in the decade. Although investigators first described the pioneering clinical use of Fluorine-18 (^18^F) sodium fluoride (NaF) as a dedicated bone-seeking imaging agent in 1962, the technological limitations of the era severely throttled its initial impact [33]. Specifically, the low sensitivity and poor spatial resolution of contemporary rectilinear scanners and early gamma cameras prevented its widespread adoption across routine clinical practice [33]. Despite these hardware limitations, during the mid-to-late 1960s, the tracer rapidly proved its worth, temporarily becoming the standard method for skeletal scintigraphy because of its exceptionally high affinity for the mineral phase of the skeleton and its high sensitivity for detecting localized alterations in bone metabolism [34].

Indeed, some of the most crippling disadvantages of imaging with the then-conventional strontium isotopes had already been successfully overcome with the introduction of ^18^F [33]. Owing to its highly favorable radiation dosimetry and remarkably fast blood clearance, clinicians found it possible to safely administer larger diagnostic activities. This translated into a significantly greater count density on the resulting images and permitted faster patient scanning speeds. For the first time, high-quality visualization of the entire human skeleton became clinically practical with ^18^F, leading it to quickly displace strontium isotopes as the scanning agent of choice, a transition backed by extensive clinical experience accumulated across several major centers [33,35,36,37,38,39]. This era of early detection was further cemented by milestone publications evaluating isotope scanning with ^18^F for the early detection of bone tumors [40].

However, this early golden era of fluoride imaging was short-lived. The widespread utility of ^18^F dictated a strict reliance on regional cyclotron production, which was highly expensive and logistically demanding at the time [41]. These economic and operational disadvantages, combined with its highly restrictive physical half-life of only 1.83 h, created what were then insoluble distribution problems regarding the nationwide commercial transport of the radionuclide. Thus, only a relatively small number of specialized medical centers positioned near a cyclotron could routinely use ^18^F, which ultimately precluded the general global acceptance of this reliable and effective radionuclide.

As a result, while [^18^F]NaF had established itself as the gold standard for bone scintigraphy in the 1960s, it was almost completely replaced and forced into a decades-long obsolescence by the commercial introduction of technetium-99m ^99^Tc-labeled diphosphonate compounds in the early 1970s [42]. These newer technetium-based agents could be easily prepared on-site via convenient, cost-effective benchtop generators, completely bypassing the distribution bottlenecks that had crippled fluorine.

The clinical resurgence of [^18^F]NaF bone imaging occurred decades later with the technological introduction and commercial maturation of Positron Emission Tomography and hybrid PET/CT imaging [42]. This advanced technology permitted the non-invasive assessment of malignant and benign bone diseases with a vastly higher diagnostic accuracy than conventional planar imaging or single-photon emission computed tomography (SPECT). This renaissance began in the late 1990s and accelerated rapidly through the 2000s, driven by a combination of profound technological changes and supply chain shifts.

Chief among these factors was the development and widespread clinical adoption of hybrid PET/CT scanners over the last two decades, which finally made it possible to routinely acquire high-quality, fully tomographic images of skeletal metabolism that were fundamentally superior to conventional SPECT [23,34]. From a diagnostic standpoint, modern [^18^F]NaF PET/CT offers significantly higher spatial resolution and contrast sensitivity than planar bone scans or SPECT, allowing radiologists to detect subtle, micro-metastatic lesions long before they manifest on traditional anatomical imaging modalities [43].

Furthermore, this methodological rebirth was accelerated by the highly favorable and rapid pharmacokinetics of the tracer. Unlike ^99m^Tc-labeled methylene diphosphonate ([^99m^Tc]Tc-MDP, see next chapter) [44,45], which requires a tedious 2-to-3-h waiting period post-injection to allow for soft-tissue blood clearance, [^18^F]NaF barely binds to serum proteins and clears from the circulation at an accelerated rate, allowing for high-contrast, diagnostic-quality imaging to be performed as soon as 30 to 60 min after initial injection [34]. This kinetic superiority became particularly vital when periodic, severe global shortages of molybdenum-99/technetium-99m (^99^Mo/^99m^Tc) generators [46,47] due to aging nuclear reactor shutdowns frequently prompted the international medical community to return to ^18^F as a logistically stable, reactor-independent alternative [43]. Today, this modern clinical availability is heavily supported by a widespread network of medical cyclotrons and advanced electronic distribution generators, allowing for the reliable, widespread commercial use of [^18^F]NaF on a global scale [48].

From a pharmacological perspective, [^18^F]NaF operates as an ideal PET bone tracer owing to its elegant uptake mechanism. Once injected, the [^18^F]NaF complex freely diffuses across capillary membranes into the extracellular fluid and is rapidly cleared from the plasma and excreted by the kidneys, leaving only about 10% of the injected diagnostic dose circulating in the blood pool one hour post-injection. This ultra-fast fluoride incorporation into the bone matrix is best modeled mathematically by a rigorous 3-part unit compartment system consisting of a distinct vascular, extravascular, and bone mineral compartment [49].

At the bone surface, the free ^18^F^−^ ions undergo a rapid, isomorphous exchange with the hydroxyl radicals (∙OH) naturally present on the surface of the hydroxyapatite crystals, chemically transforming it into fluoroapatite. Because this ^18^F substitutes directly for hydroxyl groups and covalently bonds to the exposed surfaces of newly forming bone, the localized level of [^18^F]NaF uptake depends directly on both osteogenic bone formation activity and loco-regional osteoblastic blood flow. Compared directly to traditional ^99m^Tc-diphosphonates, [^18^F]NaF is characterized by significantly faster pharmacokinetics—allowing rapid image acquisition within 30 min—and exhibits a two-fold higher absolute uptake within bone tissue, cementing its place as a modern cornerstone of molecular skeletal imaging [50].

## 4. The [^99m^Tc]Tc-MDP Era

The search for a truly satisfactory bone-scanning compound persisted with high intensity throughout the mid-20th century, prompting investigators to scrutinize a wide array of alternative radionuclides and unique chemical formulations. Early evaluations prominently featured gallium-68 (^68^Ga) [51] and technetium-99m pertechnetate ([^99m^Tc]Tc-O_4_^−^) [31,42]; concurrently, significant effort was directed toward developing complex chelates of calcium-mimetic rare-earth elements, focusing on both therapeutic and diagnostic lanthanides [30,31,42]. Yet, while these diverse alternatives initially generated substantial excitement within the academic community, their potential roles as mainstream clinical bone-scanning agents were completely and rapidly supplanted by the landmark introduction of ^99m^Tc-labeled polyphosphate in 1971 [42].

The impending clinical introduction of technetium-99m (^99m^Tc) [52], driven by the pioneering development of the commercial molybdenum-99/technetium-99m (^99^Mo-^99m^Tc) generator system [46,47], and the subsequent chemical engineering of stable bisphosphonates in the 1970s would ultimately replace early, unstable calcium mimetics as the undisputed global gold standard for skeletal imaging. This shift effectively signaled the close of the first primitive chapter in bone radiopharmaceuticals, accelerating a broad transition toward the modern era of targeted precision oncology. Indeed, the historical development of bisphosphonate-based radiopharmaceuticals represents a highly transformative journey within nuclear medicine, tracing an evolutionary path from structurally fragile phosphorus compounds to the highly sophisticated, high-affinity theranostic platforms utilized today.

This technological evolution originally began with a focused search for a resilient carrier molecule capable of effectively transporting ^99m^Tc—long considered the diagnostic “workhorse” of nuclear medicine—directly to the skeletal matrix [42]. In 1971, a foundational breakthrough was achieved when a novel bone-seeking ^99m^Tc compound formulated with sodium tripolyphosphate was introduced [42]. This milestone quickly triggered further clinical experimentation with long-chain linear polyphosphates [53] and inorganic pyrophosphate [54], which represents the simplest condensed polyphosphate consisting of two distinct phosphate moieties bridged together by a central, hydrolysable P-O-P (phosphorus–oxygen–phosphorus) chemical bond.

**Figure 2:**
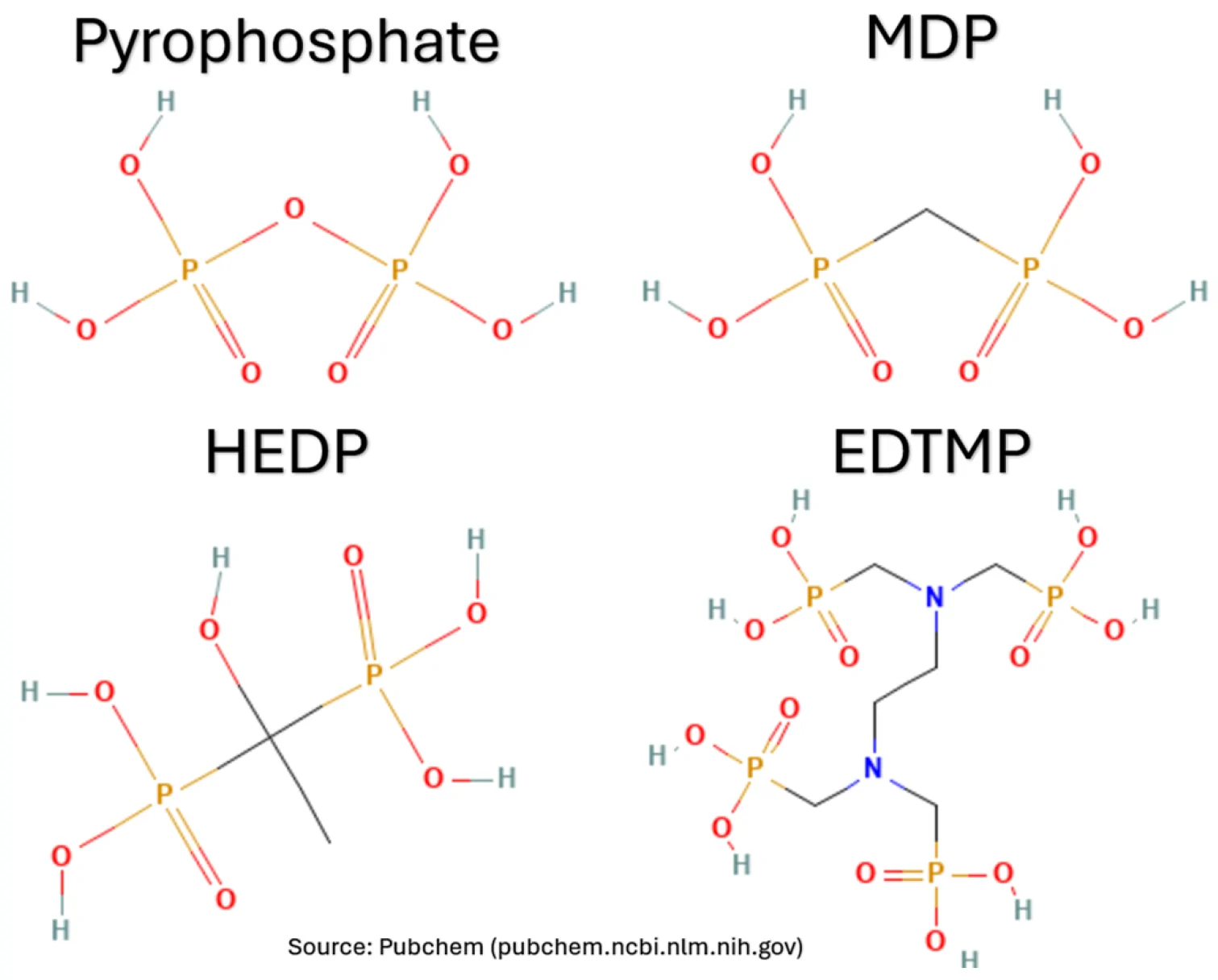
Chemical structures of some bisphosphonates.

While these early formulations successfully localized within the skeleton, they were burdened by a critical structural flaw: the central P-O-P linkage was highly susceptible to rapid enzymatic hydrolysis mediated by endogenous alkaline phosphatases circulating in the bloodstream. This rapid enzymatic breakdown triggered the premature in vivo cleavage of the tracer, releasing free technetium species into the systemic circulation, which resulted in a high soft-tissue background, severe image degradation, and poor diagnostic yield [55]. To definitively overcome this biochemical instability, researchers cleverly modified the pyrophosphate architecture by substituting the central, vulnerable oxygen atom with a highly stable carbon atom. This simple substitution yielded a rugged P-C-P (phosphorus–carbon–phosphorus) bonding sequence (Figure 2) that demonstrated high stability against enzymatic degradation, ensuring that the injected molecules could navigate the systemic circulation and reach the bone surface entirely intact [56,57].

In 1972, three independent research groups reported the successful synthesis and clinical application of the first viable bisphosphonate-based imaging agent, the [^99m^Tc]Tc- (Etidronate). Although [^99m^Tc]Tc-HEDP demonstrated significantly higher lesion-to-normal-bone contrast ratios than any of the earlier polyphosphate complexes, its absolute bone accumulation was eventually surpassed by newer, sterically optimized ligands. This limitation was likely due to spatial steric hindrance imposed by its bulky central methyl group, which slightly impeded optimal crystal binding. Mechanistically, the accumulation of all such ^99m^Tc-bisphosphonate complexes within the skeleton is fundamentally derived from the direct chemical coordination of the phosphonate groups to the exposed calcium ions residing within the hydroxyapatite mineral phase of the bone [45].

A definitive and long-lasting clinical milestone was reached in 1975 when an optimized agent, ^99m^Tc-methylene diphosphonate ([^99m^Tc]Tc-MDP), was introduced [44]. This formulation quickly established itself as the standard radiopharmaceutical for routine bone scintigraphy. The phosphonate groups of the [^99m^Tc]Tc-MDP molecule, densely populated with highly electronegative oxygen atoms, function as exceptional electron donors. This chemical property allows the central ^99m^Tc radionuclide to coordinate directly and firmly with the ligand without requiring any intermediary chemical linkers or chelating spacers [58,59,60]. Consequently, this optimized agent provided highly accelerated clearance from the vascular compartment and surrounding background soft tissues, permitting high-contrast, diagnostic-quality visualization of the entire skeletal framework within 2 to 4 h post-injection [58,59,60]. In 1980, further innovation led to the introduction of ^99m^Tc-hydroxymethylene diphosphonate ([^99m^Tc]Tc-HMDP), which strategically added a single hydroxyl group to the central carbon atom [61]. This addition facilitated the formation of a highly stable bidentate-tridentate coordination bridge with the underlying hydroxyapatite crystals, significantly enhancing the native binding affinity of the tracer compared to conventional MDP [56,57].

The clinical trajectory of contemporary bone imaging was fundamentally shaped by Subramanian’s 1971 breakthrough with ^99m^Tc-labelled polyphosphate [42], leveraging the ideal physical characteristics of ^99m^Tc [44,62], establishing the baseline for all subsequent phosphonate-based tracers. Its 6.02-h half-life and monoenergetic 140 keV gamma emission optimize imaging while reducing the radiation dose by avoiding beta decay. Combined with low cost and steady advancements in imaging device resolution [63,64], ^99m^Tc quickly became the global radionuclide of choice [62].

Despite this, notable pharmacokinetic differences exist between [^99m^Tc]Tc-MDP and positron-emitting [^18^F]NaF. Unlike [^18^F]NaF, which barely binds to serum proteins [41], [^99m^Tc]Tc-MDP displays significant plasma protein binding. This delays blood clearance, requiring a 2-to-3-h waiting interval before imaging, whereas [^18^F]NaF allows high-contrast bone scans within an hour [65]. Additionally, [^18^F]NaF is taken up by red blood cells—reaching 45% to 50% of plasma concentration (about 30% of total blood concentration)—but its free diffusibility ensures this does not interfere with skeletal accumulation [66]. In oncology, comparative trials show that [^18^F]NaF-PET is significantly more sensitive in detecting bone metastases than planar ^99m^Tc-MDP scintigraphy. While the exact contribution of the tracer itself versus the superior tomographic performance of PET instrumentation was initially difficult to separate, [^18^F]NaF-PET offers clear advantages: higher sensitivity and a shorter injection-to-scan interval, paving the way for its broader future adoption [67].

Nevertheless, [^18^F]NaF was largely replaced by ^99m^Tc-diphosphonates like [^99m^Tc]Tc-MDP in the 1970s due to contemporary limitations. Gamma cameras of that era were optimized for the 140 keV energy of ^99m^Tc and could not efficiently image the 511 keV annihilation photons of positron emitters. Furthermore, ^99m^Tc was easily accessible via commercial generators, whereas ^18^F required rare and expensive on-site cyclotrons. These logistics firmly anchored [^99m^Tc]Tc-MDP as the global standard for subsequent decades; yet, this widespread clinical reliance also forced nuclear medicine to confront the inherent chemical limitations of early technetium chemistry.

Indeed, despite anchoring routine skeletal scintigraphy for generations, these traditional technetium-diphosphonates remained far from chemically or pharmaceutically ideal. A key limitation is that agents like MDP and HMDP do not exist as well-defined, single chemical species; instead, they form complex, fluctuating mixtures of short- and long-chain oligomers, monomers, and dimers that vary depending on preparation conditions such as pH and oxygen levels [55,68]. This structural heterogeneity can directly cause variations in their biological behavior. Furthermore, their prolonged post-injection imaging window stems from a fundamental design constraint: the phosphonate groups are forced to serve a dual role, acting simultaneously as the metal-coordinating ligand for the technetium core and the Ca^2+^-binding vector for the hydroxyapatite matrix. This overlapping responsibility inevitably reduces the tracer’s inherent skeletal accumulation capacity [60,69,70].

To definitively overcome these structural and kinetic limitations, a more logical bifunctional drug design framework emerged to redefine technetium-based bone agents. This strategic approach physically decouples the bisphosphonate targeting carrier from the radiometal chelating group within the same molecule, allowing each component to function entirely independently. By conjugating stable mononuclear ^99m^Tc-chelating scaffolds (such as EC, MAG3, HYNIC or specialized tricarbonyl configurations) to an uncompromised bisphosphonate moiety, researchers successfully synthesized well-characterized, single-species radiopharmaceuticals [71,72,73]. These advanced bifunctional constructs demonstrated significantly higher hydroxyapatite binding and markedly accelerated blood clearance, yielding vastly superior bone-to-blood radioactivity ratios compared to traditional MDP [74]. This shift toward structural segregation not only offered a clear path to shorter patient waiting intervals but also established a molecular blueprint that would later bridge the gap between classic nuclear medicine and modern targeted bone therapeutics.

## 5. [^18^F]FDG: Metabolic Shift in Bone Oncology

While the [^99m^Tc]Tc-MDP era provided nuclear medicine with a robust, highly sensitive method for screening skeletal turnover, the field of bone oncology underwent a major paradigm shift with the introduction of fluorodeoxyglucose ([^18^F]FDG). Originally conceptualized as a metabolic tracer, this radiolabeled glucose analog was designed to map cerebral glucose consumption in the living human brain, thereby translating preclinical autoradiographic data—originally obtained using 2-deoxy-D-[^14^C]glucose—into a practical clinical setting [75,76]. The historic milestone for human application occurred in 1976, when the radiotracer was synthesized at the Brookhaven National Laboratory on Long Island and subsequently transported to the Hospital of the University of Pennsylvania. There, the first clinical images of a human volunteer were acquired using a prototype emission computed tomography scanner [77]. This pioneering study represented the true starting point for the clinical application of [^18^F]FDG, paving the way for the highly successful diagnostic combination of [^18^F]FDG/PET as the gold standard for detecting the elevated glycolytic activity characteristic of malignant cell populations.

As clinical interest in [^18^F]FDG grew, researchers began investigating its utility in evaluating skeletal disease, revealing a fundamental pathophysiological difference in uptake mechanisms compared to conventional bone-seeking agents. Traditional bone scintigraphy using [^99m^Tc]Tc-MDP, as well as newer [^18^F]NaF PET imaging, relies on detecting the indirect osteoblastic response and localized bone remodeling triggered by tumor-induced destruction [78,79]. Although highly sensitive, this osteoblastic targeting has historically suffered from a lack of specificity, as any benign inflammatory or traumatic process associated with increased bone turnover can mimic a metastasis [78,80,81]. In contrast, [^18^F]FDG directly measures the accelerated intratumoral glycolysis of the malignant cells themselves [78,79]. This allows [^18^F]FDG PET to potentially visualize early-stage bone marrow infiltration and tumor proliferation well before a measurable osteoblastic host reaction or structural bone destruction has had time to manifest [81,82,83,84].

Despite this clear biological advantage, the initial clinical application of [^18^F]FDG in detecting skeletal metastases faced a major limitation: the lack of precise anatomical context in standalone PET imaging [78,79]. Because early PET scanners produced functional images without structural landmarks, physicians often struggled to determine whether a suspicious area of high glucose avidity represented a true bone metastasis, an adjacent soft-tissue lesion, or a benign inflammatory process [84]. This diagnostic hurdle was successfully overcome by the development and rapid clinical integration of hybrid PET/CT scanners over the last few decades. By combining three-dimensional functional metabolic data with precise, co-registered CT anatomical localization in a single examination, hybrid imaging transformed [^18^F]FDG PET into a highly practical and efficient tool for evaluating the entire skeleton [78,84,85]. This technological leap resolved previous localization challenges, allowing clinicians to accurately differentiate intraosseous lesions from surrounding soft-tissue involvement and solidifying the role of [^18^F]FDG in bone tumor diagnostics [79,84].

In contemporary clinical practice, the diagnostic efficacy of [^18^F]FDG PET/CT in the skeleton varies significantly depending on the primary tumor type and the specific pathophysiological nature of the bone lesions. In patients with non-small cell lung cancer (NSCLC), [^18^F]FDG PET/CT is generally superior to traditional bone scintigraphy, offering much higher diagnostic accuracy (96% vs. 66%) and significantly reducing false-positive findings caused by benign, non-malignant skeletal conditions [81,82]. For breast cancer, the two modalities are often used complementarily, as [^18^F]FDG is highly sensitive for detecting early bone marrow involvement and aggressive osteolytic lesions, while conventional bone scintigraphy remains useful for identifying slow-growing, sclerotic bone metastases [80,86]. While [^18^F]FDG PET/CT demonstrates lower sensitivity for indolent, highly osteoblastic prostate cancer bone metastases due to low glycolytic activity, it provides high diagnostic value in identifying aggressive, dedifferentiated, or visceral lesions where conventional bone imaging may be inconclusive [85,87]. When evaluating primary bone tumors and sarcomas, [^18^F]FDG PET/CT is highly effective for staging Ewing sarcoma, and while historical data once favored bone scintigraphy for osteosarcoma, modern studies demonstrate that [^18^F]FDG PET/CT is more reliable for detecting skeletal lesions both at initial diagnosis and during follow-up for disease relapse [4,5,85,88].

Beyond the simple detection and localization of skeletal lesions, the clinical utility of [^18^F]FDG PET/CT extends to prognostic stratification and therapy monitoring. In primary bone tumors, the intensity of glucose metabolism, quantified through Standardized Uptake Values (SUV), directly correlates with the histological grade of the tumor, where higher metabolic activity indicates a more aggressive malignancy [4,89]. Furthermore, because changes in cellular metabolism precede structural skeletal modifications, monitoring the decline of [^18^F]FDG uptake over the course of treatment serves as a powerful surrogate marker for therapeutic response, predicting histopathological tumor response and overall patient survival [2,90]. Most recently, the imaging paradigm has continued to evolve with the introduction of dual-tracer PET/CT protocols. By combining the osteoblastic precision of [^18^F]NaF with the metabolic insights of [^18^F]FDG, this modern “one-stop-shop” imaging technique allows clinicians to comprehensively and accurately assess both skeletal remodeling and soft-tissue or marrow involvement in a single, integrated scan [85,91].

## 6. The New Beta Emitters and Their Limitations

By the [^99m^Tc]Tc-MDP era, the molecular mechanism driving the high skeletal affinity of these agents was definitively identified as chemisorption, a localized chemical process wherein the structural anions of the radiopharmaceutical directly bind to the Ca^2+^ ions exposed on the crystalline surfaces of the bone mineral matrix [56,57]. This structural uptake was characteristically found to be non-saturable and directly proportional to both regional osseous blood flow and localized osteoblastic activity, a dual mechanism that allowed clinicians to identify and map hypermetabolic metastatic “hot spots” against normal bone background.

Building upon these exact bone-seeking principles during the peak of the MDP era in the 1980s, bisphosphonate-based therapeutic agents rapidly emerged to exploit this vector. These platforms sought to deliver localized beta (β^−^) radiation directly to the microenvironment of skeletal lesions for the target-specific palliation of severe bone pain. Foremost among these developments was the synthesis of samarium-153 ethylenediamine tetramethylene phosphonic acid ([^153^Sm]Sm-EDTMP) [92], followed closely by rhenium-186 hydroxy ethylidene diphosphonate ([^186^Re]Re-HEDP) [93,94] (Figure 2), as well as early experimentation with unique Iodine-131-labeled diphosphonate complexes [95].

Parallel to these bisphosphonate-driven therapeutic breakthroughs, alternative non-bisphosphonate bone-seeking tracers and strategic elemental substitutions continued to expand the nuclear medicine toolkit. Strontium-89 chloride ([^89^Sr]SrCl_2_) was introduced and subsequently deployed for the systematic systemic treatment of diffuse skeletal metastases [96]. Rhenium-188 (^188^Re) has emerged as a valuable transition metal radionuclide in this class. As a high-energy β-emitting radioisotope obtained on-demand from a convenient, cost-effective tungsten-188/rhenium-188 (^188^W/^188^Re) generator system, ^188^Re rapidly demonstrated broad utility across a diverse variety of targeted oncology applications [97,98,99]. Moving into the 2000s, this therapeutic arsenal was further refined through the development of lutetium-177 (^177^Lu)-labeled constructs, specifically introducing ^177^Lu-EDTMP and ^177^Lu-DOTMP as advanced platforms for palliative bone pain management [100,101].

Despite the profound initial excitement surrounding these targeted beta emitters, this palliative era ultimately hit a critical clinical ceiling. While these radiopharmaceuticals successfully accumulated within malignant osseous zones, they proved therapeutically limited and could not achieve true tumor eradication due to the long tissue-penetrating range characteristic of beta particles. This extensive crossfire effect frequently extended far beyond the immediate boundary of the osteoblastic lesion, leading to severe complications within adjacent normal tissue.

In fact, from a microdosimetric perspective, profound bone marrow toxicity represents the primary dose-limiting factor across this entire modality, routinely triggering a sharp, clinically dangerous decrease in peripheral blood cell counts [16,102]. Radiation energy deposition within the hematopoietic niche is governed by continuous, low-dose-rate (LDR) exposure (typically 0.01–1.0 Gy/h) originating across four principal anatomical source compartments: the cellular marrow pool, the endosteum, the mineralized bone matrix, and circulating activity in surrounding tissues [16,102,103,104]. Because bone-seeking radiopharmaceuticals localize predominantly within skeletal tissues while emitting energetic β^−^-particles with extensive physical tissue penetration (up to several millimeters), a pronounced crossfire effect unselectively irradiates adjacent healthy hematopoietic progenitor **cells** [104,105]. Furthermore, microdosimetric dose non-uniformity across tissue and cellular micro-regions dictates that a higher mean absorbed dose is required to achieve equivalent tumor control compared to uniform exposures, further compounding off-target marrow toxicity [104,106].

Beyond spatial physical penetration and crossfire damage, the continuous low-dose-rate nature of beta emissions (0.01–1.0 Gy/h) introduces complex radiobiological phenomena, including low-dose hyper-radiosensitivity (HRS) and inverse dose-rate dynamics. At very low absorbed doses (≤1 Gy), certain tumor cell lineages (particularly micrometastatic foci and hematological precursors) fail to efficiently activate early DNA damage detection checkpoints. This creates a temporary window of hyper-radiosensitivity, rendering cells paradoxically susceptible to lethal damage before protective, adaptive DNA repair pathways are fully engaged [104]. Understanding these dose-rate kinetics is essential for optimizing therapeutic activity regimens to maximize micrometastatic cell kill while attempting to stay below the threshold for irreversible hematopoietic toxicity.

Compounding these hematological toxicities, the systemic intravenous administration of heavy beta emitters like ^89^Sr and ^186^Re was found to produce severe toxic neurological side effects in vulnerable patients, occasionally precipitating acute spinal cord neurological deficits and structural cord compression driven by localized tissue swelling and the destructive effect of radiation-induced demyelination [107].

Ultimately, the realizations that beta-emitting radiopharmaceuticals inflicted high collateral marrow damage while yielding suboptimal long-term therapeutic efficacy underscored the fundamental limitations of long-range particulate therapies. These systemic complications and clinical shortcomings created an urgent, unfulfilled need for radiopharmaceuticals with a significantly shorter, high-energy ionization footprint. This clinical bottleneck eventually paved the way for a paradigm-shifting era initiated by a study, which comprehensively redesigned the understanding of microdosimetric cellular boundaries and catalyzed the modern transition toward utilizing high-LET, short-range alpha-emitting radionuclides for targeted skeletal therapy.

## 7. The Chelators and the Theranostic Era

The technological evolution of bone-seeking radiopharmaceuticals experienced a profound paradigm shift as it transitioned from the direct, often unstable coordination of bone ligands to a highly sophisticated bifunctional drug design framework. While traditional agents like [^99m^Tc]Tc-MDP were clinically successful, they suffered from being chemically ill-defined mixtures of short- and long-chain oligomers. To definitively improve upon these structural limitations, researchers introduced a new molecular concept wherein the targeting carrier and the radiometal chelating group are separated within the same molecule, allowing each component to function independently and effectively. This bifunctional chelator (BFC) approach ensures that a stable, mononuclear complex-conjugated ligand leaves the inherent biological affinity of the bisphosphonate carrier entirely uncompromised by the presence of the radiometal [72,74].

By establishing a platform where the chelating moiety could be swapped without altering the targeting vector, this design strategy directly facilitated the rise of the modern theranostic paradigm. This revolution was accelerated by the introduction of the commercial ^68^Ge/^68^Ga generator system [108], which provided on-demand, cost-effective availability of a positron-emitting isotope without the clinical burden or expense of an on-site cyclotron. Leveraging this generator, modern structural evolution focused heavily on macrocyclic bisphosphonates that utilize highly stable chelators like DOTA or NOTA to firmly bind trivalent radiometals. A prominent milestone within this framework was the development of [^68^Ga]Ga-BPAMD, a tracer that demonstrated exceptional bone accumulation and provided high-resolution PET/CT images of osteoblastic metastases that matched the structural clarity of native [^18^F]NaF [109]. Building upon this success, researchers synthesized [^68^Ga]Ga-DOTAZOL, an investigational DOTA-conjugated zoledronate derivative that demonstrated high binding affinity to the hydroxyapatite matrix and favorable target-to-background contrast in early clinical evaluations [110].

In a parallel avenue of development, the robustness of the macrocyclic bifunctional strategy was evaluated using complex chelates of rare-earth elements, primarily exploring therapeutic and diagnostic lanthanides such as Samarium-153 (^153^Sm), Holmium-166 (^166^Ho), and Lutetium-177 (^177^Lu) [31]. Early generations of systemic radionuclide therapies, such as [^153^Sm]Sm-EDTMP (Quadramet) and configurations using N-hydroxy ethylene diamine triacetic acid (HEDTA) ligands, relied on open-chain, acyclic phosphonate structures [92,111]. However, the field rapidly advanced toward macrocyclic phosphonate chelates like DOTMP, which form highly rigid, kinetically inert complexes with +3 metal cations. This structural rigidity drastically reduced the risk of off-target radiometal dissociation in vivo, protecting healthy organs while maintaining maximum affinity for the bone mineral phase. Within this lanthanide group, ^177^Lu emerged as a therapeutic cornerstone owing to its favorable 6.7-day physical half-life and moderate-energy beta emission (E_β,max_ = 0.497 MeV). Although its shorter mean tissue penetration range (∼0.67 mm) efficiently targets microscopic tumor deposits compared to higher-energy beta emitters, crossfire irradiation to the adjacent hematopoietic red marrow remains significant, rendering hematologic toxicity an important clinical consideration [112]. With stable macrocyclic scaffolds established, the current frontier of nuclear oncology has achieved its most advanced iteration by deploying matched radionuclide pairs on identical molecular vectors to execute a true “see and treat” theranostic strategy. A promising theranostic concept relies on matched ^68^Ga/^177^Lu radionuclide pairs, which can be extended to bone-seeking bisphosphonates (such as the investigational [^68^Ga]Ga-/[^177^Lu]Lu-DOTAZOL pair) to evaluate skeletal lesions prior to targeted therapy [113]. Similarly, the field leverages the ^99m^Tc/^188^Re theranostic pair [114,115]. By exploiting the nearly identical chemical and coordination properties shared between technetium and rhenium, clinicians can use widely accessible ^99m^Tc-labeled diagnostic agents to accurately predict the in vivo biodistribution, clearance kinetics, and internal dosimetry of high-energy, therapeutic ^188^Re-labeled analogs like HEDP or DMSA [98,116].

Crucially, as this era matured, nuclear medicine broke free from the historical constraints of the bisphosphonate backbone entirely. Rather than targeting the secondary bone reaction—the metabolic, osteoblastic remodeling triggered by the lesion—the focus shifted toward molecularly targeting the primary tumor cells residing within the metastatic bone microenvironment. This breakthrough is exemplified by the introduction of Prostate-Specific Membrane Antigen (PSMA) theranostics. By utilizing the small-molecule ligands PSMA-11 and PSMA-617, the exact same cellular target can be imaged with ^68^Ga or treated via radionuclide therapy with ^177^Lu, enabling the highly selective eradication of prostate cancer cells directly within the marrow spaces. Mirroring this cell-directed success, [^68^Ga]Ga-FAPI-04 (Fibroblast Activation Protein Inhibitor) was developed to target the cancer-associated fibroblasts within the tumor stroma. In head-to-head clinical comparisons, [^68^Ga]Ga-FAPI-04 demonstrated superior diagnostic sensitivity for detecting bone metastases across a wide spectrum of malignancies compared to conventional [^18^F]FDG, successfully identifying 205 skeletal lesions compared to a mere 146 in a single cohort study [7,8]. Recent structural modifications (such as albumin-binding moieties ([^177^Lu]Lu-EB-FAPI, [^177^Lu]Lu-FAPI-C16) and homodimeric designs ([^177^Lu]Lu-DOTAGA-FAPI) substantially extend systemic circulation and tumor retention as well, further enhancing therapeutic response [117]. By synthesizing single-species bifunctional designs, matched radionuclide pairs, and cell-specific targeting vectors, this evolutionary path allows diagnostic imaging to closely estimate therapeutic biodistribution and target uptake, paving the way toward individualized precision oncology in the management of advanced bone disease. Innovative theranostic radionuclide pairs offer distinct physical advantages: terbium-161 delivers localized Auger electron radiation to micrometastasized lesions, lead-212 acts as an in vivo alpha generator, and copper-64/67 sarcophagine complexes (Cu-Sar-bisPSMA) provide chemically identical PET/RPT matching [117]. Also, theranostic targeting of bone metastases is expanding beyond PSMA and FAPI toward novel antigens, including Nectin-4 ([^68^Ga]Ga-N188), uPAR ([^177^Lu]Lu-AE105), and integrin αvβ6 ([^68^Ga]Ga-Trivehexin), which enable selective lesion detection with reduced background accumulation [117].

## 8. The Paradigm-Shifting Alpha Mimetic and the Targeted Alpha Therapy

While the introduction of medium-energy β^−^-emitters represented a significant milestone in palliative skeletal oncology, their clinical efficacy remains fundamentally constrained by physical properties (Table 1 and Table 2). The relatively long tissue penetration range of β^−^-particles combined with their low linear energy transfer (LET) leads to a substantial “cross-fire” effect, irradiating adjacent normal haematopoietically active bone marrow and frequently causing severe myelosuppression that restricts dose escalation. Consequently, a considerable subset of patients exhibits suboptimal treatment response or eventual disease progression [118,119,120,121]. To overcome these physical limitations, targeted alpha therapy (TAT) emerged as a revolutionary alternative. Alpha particles possess a high LET and an extremely short tissue range (50–100 µm), corresponding to merely a few cell diameters. This localized energy deposition generates dense track ionization that induces complex, non-repairable double-strand DNA breaks within tumor cell nuclei [122,123], and stimulates immunogenic cell death via the cGAS-STING-NLRP3 pathway, resulting in pyroptosis and DAMP release recruit CD8+ T cells to skeletal lesions [117]. When targeted to bone surfaces, the short path length of α-particles drastically reduces cross-fire radiation into the underlying bone marrow compared to approved β^−^-emitters, producing potent cytotoxicity while sparing healthy hematopoietic tissue and demonstrating therapeutic activity even in β^−^-resistant disease [9,120,121,124,125]. However, while shorter particle ranges theoretically minimize collateral damage to adjacent non-target tissues, particle range alone does not automatically guarantee bone marrow sparing. Hematological toxicity is ultimately governed by a multifactorial interplay, including systemic biodistribution kinetics, blood clearance rates, target localization precision, lesion geometry, administered activity, and the potential redistribution of radioactive decay daughters.

This microdosimetric trade-off highlights distinct radiobiological paradigms within the targeted tumor microenvironment. While short-range alpha emitters (and Auger electrons) deliver ultra-dense energy deposition strictly confined to target-binding tumor cells (thereby sparing underlying hematopoietic bone marrow), their restricted emission radius may limit direct radiation delivery to non-binding stromal components or heterogeneous, target-negative cell clusters. Conversely, longer-range beta emitters rely heavily on cross-fire radiation to cover surrounding tumor stroma and unlabeled neoplastic cells, a mechanism complemented by intercellular bystander effects, where irradiated cells transmit paracrine stress signals (e.g., reactive oxygen species and cytokines) to induce cytotoxicity in adjacent non-irradiated cells [104].

The first paradigm-shifting clinical breakthrough in intrinsic, bone-seeking α-therapy came with radium-223 dichloride ([^223^Ra]RaCl_2_). Acting as a natural calcium mimetic, ^223^Ra targets the skeleton natively without requiring a targeted carrier ligand, forming complexes with the bone mineral hydroxyapatite specifically in regions of heightened osteoblastic bone turnover characteristic of osseous metastases [126]. The landmark Phase III ALSYMPCA trial evaluated [^223^Ra]RaCl_2_ in 921 patients with metastatic castration-resistant prostate cancer (mCRPC) and symptomatic bone metastases, demonstrating a statistically significant improvement in median overall survival (14.9 months vs. 11.3 months in the placebo group; HR = 0.70, *p* < 0.001) alongside prolonged times to alkaline phosphatase (ALP) and prostate-specific antigen (PSA) progression [126]. Monte Carlo phantom dosimetry studies (e.g., using MCNP algorithms) and microdosimetric comparisons have further validated this physical advantage: the bone-absorbed dose per disintegration from α-particles of [^223^Ra]RaCl_2_ is approximately 24 times higher than that from [^89^Sr]SrCl_2_, while conventional β^−^-emitters like [^188^Re]Re-HEDP exhibit up to 40-fold higher absorbed doses to the sensitive bone marrow [127,128,129]. These favorable findings led to the United States Food and Drug Administration (FDA) approval of [^223^Ra]RaCl_2_ in 2013 [126,128].

Despite its proven survival benefit, [^223^Ra]RaCl_2_ faces notable clinical, safety, and economic challenges. During the radioactive decay cascade of ^223^Ra, short-lived α-emitting daughter progeny (lead-211 and bismuth-211) are generated; these recoiling daughters can dissociate from the bone matrix and redistribute to healthy organs, posing systemic toxicity risks [130]. Furthermore, safety concerns intensified following clinical trials evaluating combination regimens; specifically, co-administering [^223^Ra]RaCl_2_ with second-generation antiandrogens like abiraterone acetate resulted in a significantly increased incidence of early deaths and non-metastatic bone fractures. These findings prompted regulatory restrictions from the FDA and EMA, mandating the use of concurrent bone-protective agents (such as bisphosphonates or denosumab) and restricting unsafe combination protocols [130]. In addition to toxicity concerns, the prohibitively high manufacturing and procurement costs of [^223^Ra]RaCl_2_ present a major obstacle to its widespread global implementation.

To expand the scope of α-therapy beyond intrinsic calcium mimetics, modern nuclear medicine has leveraged stable chelation chemistry—building directly upon the “Theranostic Era”—to attach α-emitters to highly specific tumor-targeting ligands. Among these, actinium-225 (^225^Ac) has emerged as a particularly promising radionuclide due to its favorable 10-day physical half-life and a decay cascade yielding four net α-particles, which delivers high localized relative biological effectiveness (RBE)to micrometastatic foci. Utilizing DOTA-compatible chelating platforms, investigational bone-targeted radiopharmaceuticals such as [^225^Ac]Ac-DOTAZOL have shown potent therapeutic activity in preclinical and early clinical studies when compared with diagnostic ([^68^Ga]Ga-DOTAZOL) and β^−^-emitting ([^177^Lu]Lu-DOTAZOL) counterparts, provided that strategies to mitigate renal toxicity are implemented [131]. Concurrently, receptor-targeted alpha therapies aimed at cell-surface antigens have gained immense traction. Notably, PSMA-targeted ligand therapy using [^225^Ac]Ac-PSMA-617 demonstrated remarkable, complete clinical and radiologic responses in patients with advanced, heavy bone-tumor burdens who were completely refractory to standard therapies and β^−^-radioligands [9]. Comparative cellular biokinetic and Monte Carlo modeling reveals that [^225^Ac]Ac-PSMA delivers a 900-fold higher radiation absorbed dose to prostate cancer cell nuclei than [^177^Lu]Lu-iPSMA and a 14-fold higher dose than [^223^Ra]RaCl_2_ per unit of activity retained in bone, solidifying targeted α-therapy as a superior modality for metastatic bone disease [6].

However, while the 50–100 µm path length of α-particles is ideal for cell-cluster destruction, achieving ultimate subcellular damage directly at the sub-cellular or genomic level requires exploring radionuclides with even shorter radiation ranges, leading logically to the molecular targeting of Auger electron emitters.

## 9. Molecular Targeting of Auger Electron Emitters

**Figure 3:**
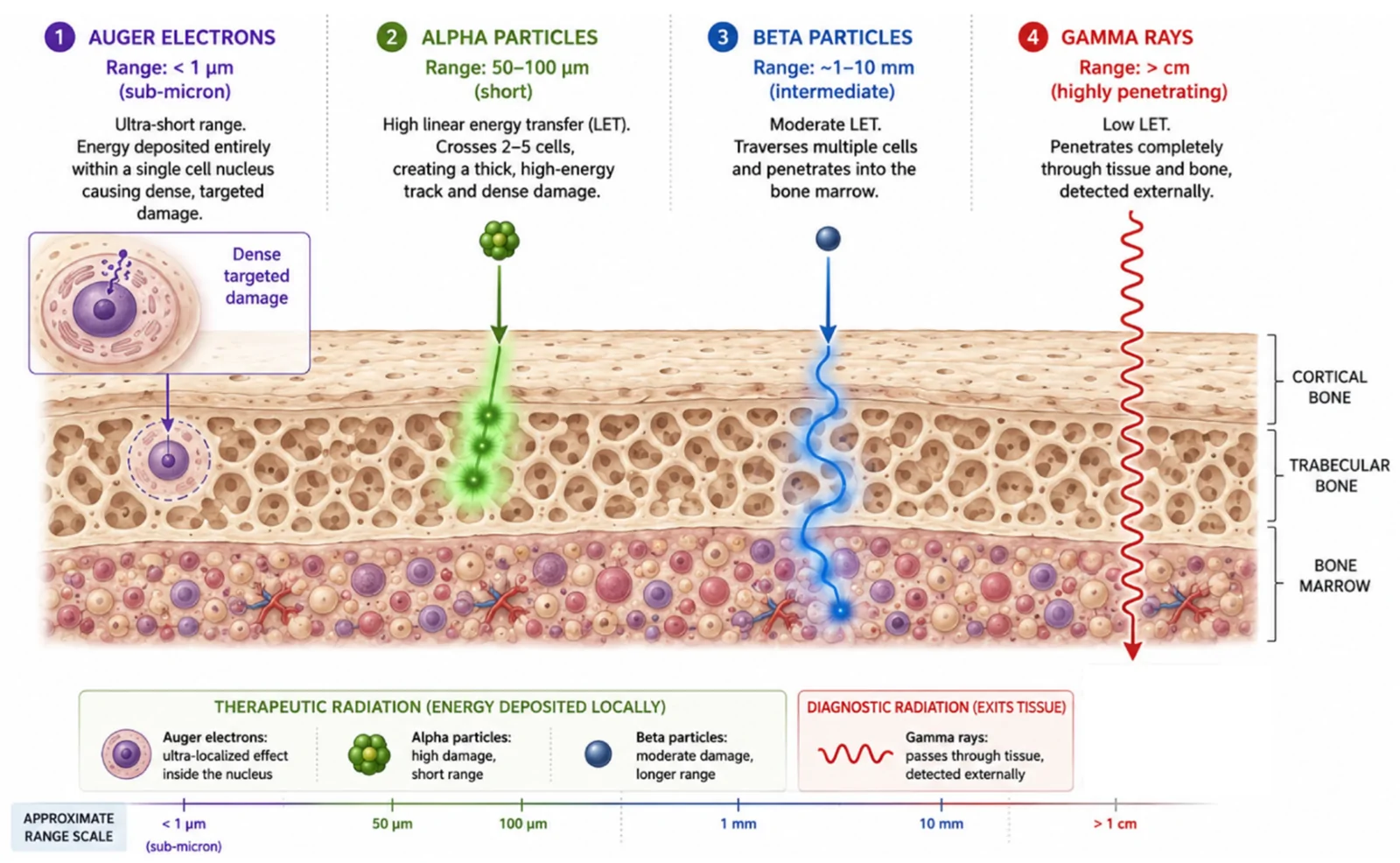
Tissue penetration depth in bone. Therapeutic rationale: High local energy deposition (High LET) within a short range (α, Auger, β^−^) minimizes collateral bone marrow damage while maximizing tumor kill. Diagnostic rationale: High penetration (γ) allows radiation to escape the body for external detection.

While α-particles deliver high linear energy transfer (LET) across several cell diameters (50–100 µm), Auger electron (AE) emitters refine spatial energy deposition to the nanometer scale (<0.5 µm) [132,133] (Figure 3). First described by Lise Meitner and Pierre Auger, this phenomenon originates from decay via electron capture or internal conversion [134,135]. The resulting inner atomic shell vacancy triggers a cascade of electronic transitions, ejecting multiple low-energy electrons per nuclear decay (typically 4 to 37 AEs with energies < 25 keV) [136]. Achieving optimal therapeutic efficacy with such ultra-short-range radiation requires a fundamental consideration of sub-cellular microdosimetry and active cellular uptake mechanisms [104,137,138]. Because the tissue range of AEs is restricted to sub-micrometer scales (<1 μm), energy deposition is densely concentrated, delivering a high localized linear energy transfer (LET of 4–26 keV/μm) that substantially surpasses that of conventional β−-particles (∼0.2 keV/μm) [104,133,136,137]. Beyond high localized LET, several clinically prominent Auger electron emitters (such as ^125^I and ^111^In) decay into stable daughter isotopes, minimizing long-term residual radiation burden from secondary decay chain products [136]. Additionally, because the biological effectiveness of AEs requires close proximity to nuclear DNA, radiopharmaceuticals circulating in the bloodstream or bone marrow exhibit minimal non-target cytotoxicity [139]. Concomitant γ-photon emissions frequently allow real-time single-photon emission computed tomography (SPECT) imaging, facilitating true theranostic integration [136]. Importantly, while historical bone palliation required modest radiation doses for symptomatic relief, inhibiting progressive bone tumor growth demands significantly higher localized absorbed doses, a requirement uniquely suited to high-LET sub-cellular emitters [132]. Furthermore, this strict microdosimetric spatial requirement provides intrinsic bone marrow sparing: non-internalized constructs circulating in plasma or extracellular marrow fluids exert minimal cytotoxicity to hematopoietic stem cells [104,136,137].

Bridging the gap between medium-range particles and nanometer-scale emitters, tin-117m (^117m^Sn) emits short-range conversion electrons (CE) with a tissue penetration of 220–290 µm (~10 cell layers) [140]. Formulated in its optimal +4 oxidation state as [^117m^Sn]Sn(IV)-DTPA, it targets bone surfaces with high avidity [141]. Preclinical dosimetry using mouse marrow biologic dosimeters demonstrated up to an 8-fold marrow-sparing therapeutic advantage for [^117m^Sn]Sn(IV)-DTPA over energetic β^−^-emitters such as [^32^P]orthophosphate [140]. Clinically, Phase I/II trials confirmed that [^117m^Sn]Sn(IV)-DTPA provided complete pain relief in 25% of treated patients and >50% pain reduction in an additional third, with a mean response duration of 3.3 months [142]. Nevertheless, clinical adoption has been hampered by low bone affinity compared to newer bisphosphonates and the susceptibility of tin to dissociate from the DTPA complex [132,141].

To capitalize fully on the dense cytotoxicity of true AEs, passive surface chemisorption is insufficient; targeted radiopharmaceuticals must undergo active receptor-mediated endocytosis and nuclear translocation to position the radionuclide within close proximity (<50 Å) to genomic DNA for non-repairable double-strand break induction [138]. Early AE constructs like [^111^In]In-pentetreotide produced minimal tumor regression because they failed to translocate efficiently to the cell nucleus, leading to their clinical replacement by β^−^-emitting [^90^Y]Y-DOTATOC and [^177^Lu]Lu-DOTATATE [143,144,145]. Conversely, internalizing targeted agents such as [^111^In]In-EGF demonstrate substantial therapeutic efficacy against EGFR-expressing metastases because EGFR expression is minimal in bone marrow stem [139,146,147,148]. Advancing sub-cellular targeting even further, radiolabeled poly(ADP-ribose) polymerase inhibitors (PARPi), such as [^123^I]I-MAPi, exploit “PARP trapping” [149,150,151,152]. This mechanism docks the AE-emitting radioisotope within 50Å of DNA during active repair, delivering concentrated ionizing tracks that induce lethal double-strand breaks [149,151]. Preclinical models demonstrated that [^123^I]I-MAPi achieves rapid tumor internalization, remarkable tumor-to-background contrast, and significant survival prolongation without significant off-target toxicity [149,153], a paradigm supported by first-in-human PET imaging using [18F]F-PARPi (NCT04173104) [154].

To circumvent the strict dependence of pure Auger emitters on intimate nuclear proximity, hybrid radionuclides such as terbium-161 (^161^Tb) have gained prominence. With a 6.9-day half-life, ^161^Tb co-emits medium-range β^−^-particles alongside a high flux of short-range AEs and CEs [155,156]. This combined emission profile enables the eradication of larger tumor masses via β^−^ cross-fire while simultaneously destroying single micrometastatic cells via local AE intensity, proving therapeutically superior to ^177^Lu in comparative DOTA-chelated preclinical studies [155,156]. Other emerging AE candidates include ^201^Tl (emitting an exceptional 36.9 AEs per decay via Na^+^/K^+^ pump uptake) [157,158,159,160], ^197m^/^197^Hg, ^119^Sb (t1/2 = 1.6d; ~24AEs/decay), although production constraints currently limit their widespread clinical translation [161,162,163].

In skeletal targeted therapy, osteophilic transition metal complexes offer a compelling strategy. Radioactive platinum isotopes (^195^Pt) conjugated to bisphosphonate ligands ([^195m^Pt]Pt-BP) combine bone-mineral affinity with direct DNA intercalation upon complex disintegration, delivering an 11-fold enhancement in cytotoxicity over non-radioactive counterparts [161,164,165,166]. Similarly, palladium isotopes (^103^Pd and ^109^Pd) coordinated with bisphosphonates and aromatic co-ligands (e.g., bipyridine or phenanthroline) exhibit strong hydroxyapatite binding and potent cell killing in metastatic prostate and ovarian carcinoma lines [167,168,169,170]. Crucially, these palladium systems can operate as in vivo radiotherapeutic generators (^103^Pd/^103^mRh and ^109^Pd/^109m^Ag), releasing short-lived, highly cytotoxic AE/CE-emitting daughter nuclides directly within the osseous microenvironment to achieve sustained, localized tumor control [167,170].

## 10. Molecular Mechanisms of Bone Radiopharmaceuticals

Healthy bone homeostasis is strictly maintained through a tightly regulated dynamic equilibrium between osteoclastic bone resorption and osteoblastic bone formation. Central to this homeostatic control is the receptor activator of nuclear factor-κB (RANK), its cognate ligand (RANKL), and the soluble decoy receptor osteoprotegerin (OPG). Osteocytes, osteoblasts, and bone marrow stromal cells produce RANKL, a transmembrane or soluble cytokine belonging to the TNF superfamily. RANKL binds to RANK receptors expressed on the surface of hematopoietic osteoclast precursors, triggering intracellular signaling cascades, primarily mediated via TRAF6 recruitment, NF-κB activation, and subsequent upregulation of the master osteoclastogenic transcription factor NFATc1 (nuclear factor of activated T-cells c1). In the presence of macrophage colony-stimulating factor (M-CSF), this signaling cascade drives pre-osteoclast commitment, cell-fusion, differentiation, survival, and bone-resorbing activity. Conversely, OPG, a soluble decoy receptor secreted by osteoblasts and stromal cells, acts as a physiological brake by sequestering RANKL with high affinity, preventing its interaction with RANK and thereby suppressing osteoclastogenesis [1,23].

In the setting of metastatic bone disease, disseminated tumor cells infiltrate the endosteal niche and severely disrupt this delicate balance, establishing a self-sustaining pathological feedback loop known as the metastatic “vicious cycle”. Upon colonizing the bone microenvironment, malignant cells secrete key humoral factors—most notably parathyroid hormone-related protein (PTHrP), interleukin-6 (IL-6), and tumor necrosis factor-alpha (TNF-α). These paracrine signals markedly upregulate RANKL expression while simultaneously suppressing OPG synthesis in adjacent osteoblasts and stromal cells. The resulting excess of uninhibited RANKL signaling induces profound, hyperactive osteoclastogenesis and accelerated bone destruction [1,23].

As the mineralized bone matrix is progressively degraded by hyperactive osteoclasts, immobilized growth factors embedded within the matrix—including transforming growth factor-beta (TGF-β), insulin-like growth factors (IGFs), and fibroblast growth factors (FGFs)—are released directly into the microenvironment. These matrix-derived factors subsequently bind to specific receptors on tumor cells, further promoting malignant cell survival, proliferation, and persistent PTHrP release, thereby perpetuating the osteolytic cascade [1,23]. Therapeutically, targeted inhibition of this axis using the monoclonal antibody denosumab (which directly neutralizes RANKL) breaks this destructive loop, providing a synergistic bone-protective mechanism frequently combined with endoradiotherapy (e.g., ^223^Ra or ^177^Lu radiopharmaceutical regimens) to reduce skeletal-related events (SREs) [1,23].

Conversely, in osteosclerotic (osteoblastic) metastases, such as those predominantly originating from prostate cancer, tumor-derived factors (e.g., endothelin-1, Wnt signaling proteins, and bone morphogenetic proteins) drive uncoupled, chaotic osteoblastic bone deposition. This aberrant mineral exchange generates localized zones of intense hydroxyapatite turnover. Targeted radiopharmaceuticals—whether exploiting surface chemisorption to hydroxyapatite (e.g., [^99m^Tc]Tc-MDP), alkaline earth calcium mimicry (e.g., ^223^Ra^2+^, ^89^Sr^2+^), or direct cell-surface biomarker uptake—leverage this heightened local metabolic activity within the vicious cycle to deliver targeted micro-dose ionizing radiation precisely to the tumor–host interface [24,56,57].

**Figure 4:**
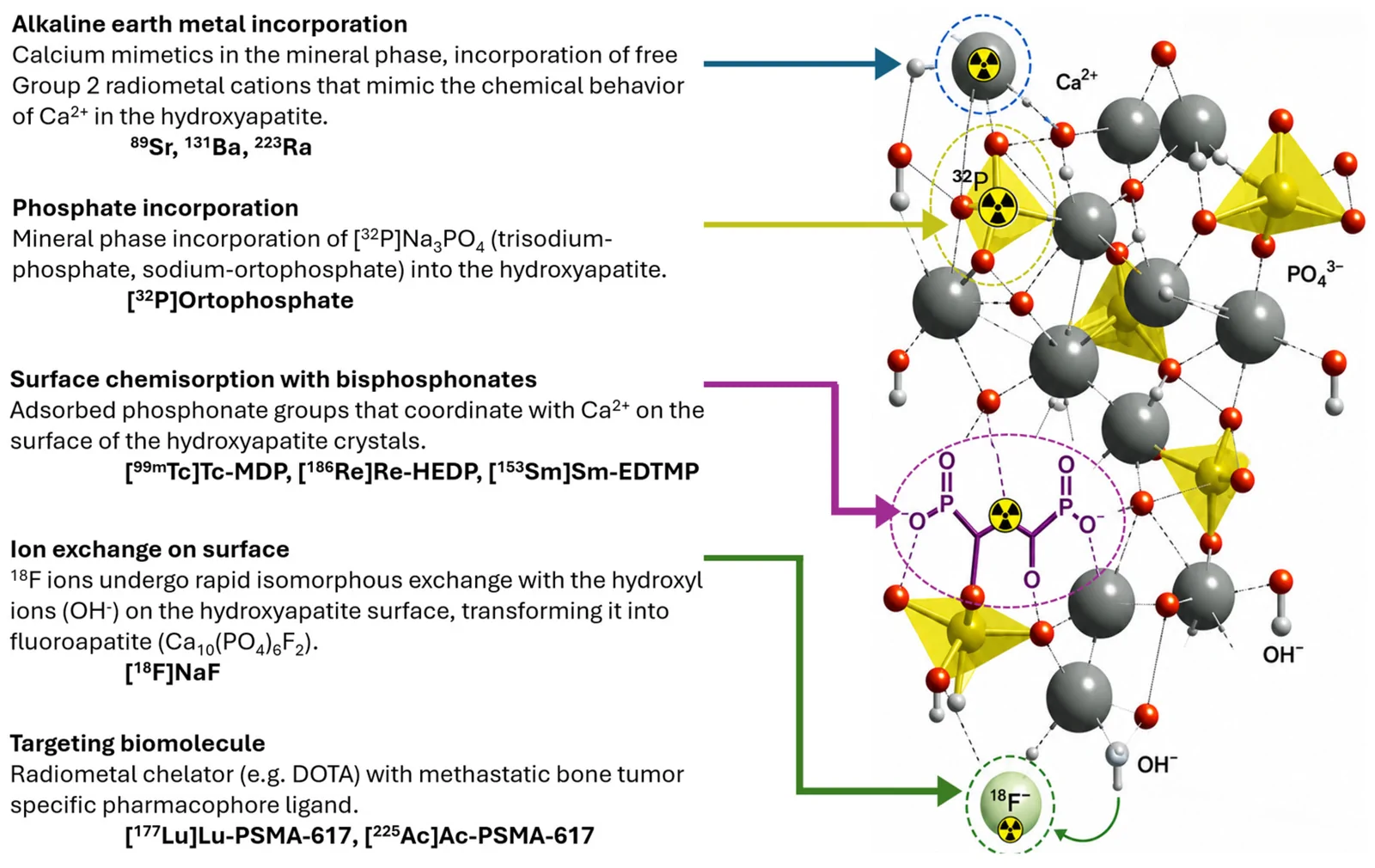
Pharmaceutical mechanisms of bone radiopharmaceuticals in hydroxyapatite *(The 3D crystal structure of hydroxyapatite was adapted from ChemTube3D, University of Liverpool)* [171].

At the biochemical level of bone remodeling, osteoclasts resorb mineralized bone while osteoblasts deposit an organic osteoid matrix predominantly composed of type I collagen. Subsequent mineralization requires the orderly deposition of calcium and phosphate ions to yield crystalline hydroxyapatite (Ca_10_(PO_4_)_6_(OH)_2_). A pivotal enzyme governing this cascade is alkaline phosphatase (ALP), which hydrolyzes inorganic pyrophosphate—a potent endogenous inhibitor of calcification—into inorganic phosphate ions, thereby establishing a permissive biochemical environment for hydroxyapatite crystallization. Based on their primary target interactions, chemical properties, and subcellular fates, bone-targeted radiopharmaceuticals operate through five distinct molecular pathways (Figure 4) [56,59].

### 10.1. Alkaline Earth Metal Incorporation (Calcium Mimetics)

The most direct mechanism for targeting mineralizing bone relies on radionuclides that function as structural calcium mimetics. Group 2 alkaline earth metals—such as strontium (Sr^2+^), barium (Ba^2+^), and radium (Ra^2+^)—share valence electron configurations and ionic radii similar to calcium (Ca^2+^). Administered as free divalent cations, these radionuclides seamlessly integrate into the expanding hydroxyapatite crystal lattice during active osteoblastic mineralization. Often classified as “volume seekers,” they deposit throughout the newly mineralized matrix, particularly within osteosclerotic metastatic lesions. Strontium-89 (^89^Sr) and radium-223 (^223^Ra) represent the primary clinical agents operating via this pathway. Notably, ^223^Ra introduced a paradigm shift as the first targeted α-emitter to demonstrate an overall survival benefit in metastatic castration-resistant prostate cancer (mCRPC) by delivering high-LET radiation to bone surfaces while preserving hematopoietic progenitor cells. Barium-131 (^131^Ba) is currently being evaluated as a diagnostic SPECT match for radium therapy [3,25,121,126].

### 10.2. Phosphate Incorporation

Unlike surface-binding agents, inorganic phosphate radiopharmaceuticals incorporate directly into the core matrix of the hydroxyapatite lattice as structural anions. Following administration as trisodium orthophosphate, phosphorus-32 ([^32^P]orthophosphate) mimics endogenous inorganic phosphate. Driven by local ALP activity during osteoblastic matrix formation, ^32^P is actively utilized in the synthesis of new hydroxyapatite crystals (PO_4_^3−^ site substitution). Because it penetrates the entire depth of newly forming mineral layers, ^32^P acts as an ionic volume seeker. Although historically pivotal as one of the earliest systemic bone radiopharmaceuticals for myeloproliferative disorders and osseous palliation, its clinical utility has waned due to significant myelosuppression resulting from the high beta-particle energy and cross-fire irradiation to adjacent bone marrow [14,16,17,18,21].

### 10.3. Surface Chemisorption with Bisphosphonates

In contrast to lattice incorporation, phosphonate-based complexes interact exclusively with the exposed outer layer of bone mineral. This process occurs via chemisorption—a surface interaction characterized by coordination bonds between the oxygen atoms of the phosphonate groups (P=O and P-O^−^) and accessible Ca^2+^ cations on the hydroxyapatite crystal surface. Polyphosphonates and bisphosphonates (containing a stable P-C-P backbone) exhibit rapid blood clearance and marked avidity for active skeletal mineralization fronts. Introduced in the 1970s, technetium-99m methylene diphosphonate ([^99m^Tc]Tc-MDP) established the clinical standard for SPECT bone scintigraphy. Therapeutic radiometals conjugated to polyphosphonate ligands—such as rhenium-186 HEDP ([^186^Re]Re-HEDP) and samarium-153 EDTMP ([^153^Sm]Sm-EDTMP)—exploit this same surface coordination mechanism to deliver localized β^−^-radiation for bone pain palliation [44,56,57,69,92,93].

### 10.4. Ion Exchange on Surface (Fluoroapatite Formation)

Fluoride ions operate through a unique mechanism of isomorphous surface exchange. Following intravenous administration of sodium fluoride ([^18^F]NaF), free [^18^F]F^−^ ions passively diffuse through the capillary membrane into the hydration shell surrounding hydroxyapatite crystals. Due to near-identical ionic radii and electronegativity, [^18^F]F^−^ rapidly undergoes a one-to-one exchange with surface-exposed hydroxyl ions (OH^−^), converting the surface mineral phase into fluoroapatite (Ca_10_(PO_4_)_6_F_2_). [^18^F]NaF PET offers significantly faster blood clearance, higher bone-to-background contrast, and superior spatial resolution compared to conventional diphosphonate SPECT agents [33,34,36,37,43,49].

### 10.5. Targeted Radioligand Therapy (Precision Cellular Targeting)

Representing a fundamental evolution in nuclear oncology, targeted radioligand therapy shifts the therapeutic target from the bone’s reactive stromal microenvironment to biomarker-expressing malignant cells residing within the marrow compartment. This mechanism utilizes a high-affinity targeting vector (e.g., small-molecule PSMA inhibitors) conjugated via a bifunctional chelator (e.g., DOTA, Macropa) to a therapeutic or diagnostic radionuclide. Radioligands such as [^177^Lu]Lu-PSMA-617 or [^225^Ac]Ac-PSMA-617 bind directly to receptors overexpressed on tumor membranes, delivering destructive ionizing radiation directly to target cells while sparing surrounding mineralized tissue and healthy marrow. This mechanism underpins the theranostic framework, enabling diagnostic PET imaging (e.g., with [^68^Ga]Ga-PSMA-11) to confirm target expression prior to administering radionuclide therapy. Emerging macrocyclic bisphosphonate hybrids, such as DOTAZOL, integrate this modern chelation chemistry with traditional skeletal chemisorption, offering dual bone-targeting and theranostic radiometal delivery [6,7,110,113,131]. Beyond structural affinity, the cellular internalization and accumulation kinetics of these radioligands introduce a critical radiobiological determinant known as the uptake-rate effect. From a radiobiological perspective, slower systemic accumulation or delayed target binding allows tumor cells to mount adaptive survival responses—such as the upregulation of enzymatic DNA repair pathways—during the initial low-dose-rate exposure phase. This adaptive process flattens the shoulder of the cell survival curve, thereby diminishing net therapeutic cytotoxicity compared to rapid, high-affinity target uptake [104].

## 11. Conclusions, Trends, Future Perspectives

The nearly century-long trajectory of bone radiopharmaceuticals reflects one of the most dynamic evolutionary journeys in nuclear medicine and clinical oncology. What began in the early 20th century as an accidental discovery of bone contamination in dial painters—and evolved into crude palliative interventions using unsealed sources—has today matured into a highly sophisticated, cell-directed theranostic discipline. A critical analysis of this historical continuum reveals several overarching trends that define the present landscape and delineate the future directions of skeletal nuclear oncology.

### 11.1. The Symbiosis of Radiochemistry and Imaging Hardware

A key lesson from the history of bone radiopharmaceuticals is that the clinical utility of a radioisotope is fundamentally bounded by the capabilities of contemporaneous imaging instrumentation. The dramatic narrative of fluorine-18 ([^18^F]NaF) exemplifies this co-evolution. Although [^18^F]NaF demonstrated superior bone kinetics as early as the 1960s, hardware limitations forced it into a multi-decade obsolescence in favor of generator-produced ^99m^Tc-diphosphonates like [^99m^Tc]Tc-MDP. It was only with the technological maturation and widespread clinical deployment of hybrid PET/CT and PET/MR instrumentation—coupled with molybdenum-99 supply vulnerabilities—that [^18^F]NaF experienced its remarkable modern renaissance. Future radiopharmaceutical development will similarly depend on advancements in total-body PET systems and ultra-high-resolution digital SPECT, enabling unprecedented sensitivity for micro-metastatic detection and dynamic kinetic modeling.

### 11.2. Democratization and Diversification of the Radionuclide Toolkit

Rather than consolidating around a few standard isotopes, nuclear medicine continues to expand its radionuclide arsenal. Innovations in nuclear physics, high-current cyclotrons, linear accelerators, and clinical-grade radionuclide generators (such as ^68^Ge/^68^Ga and ^188^W/^188^Re) have decentralized isotope access. This technological democratization has enabled the translation of novel radiometals and unconventional decay modes into routine research. From short-lived positron emitters to emerging Auger electron and conversion electron sources (^161^Tb, ^117m^Sn, ^195m^Pt), the expanding toolkit ensures that physical decay characteristics can be tailored precisely to specific tumor phenotypes and anatomical scales. From an economic standpoint, while the synthesis and logistical management of short-lived therapeutic radionuclides present supply-chain challenges, integrated radiotheranostics offer long-term cost-effectiveness. By providing rapid diagnostic stratification, theranostics prevents prolonged, ineffective systemic treatments and reduces overall healthcare burdens through targeted intervention [172].

### 11.3. The Precision Paradigm Shift: Range, Theranostics, and Cellular Targeting

The transition toward personalized precision oncology in skeletal nuclear medicine is underpinned by three converging conceptual shifts. First, driven by advanced Monte Carlo simulations and radiobiological discoveries, therapeutic strategies have undergone a radical microdosimetric range refinement. Clinical protocols have progressively transitioned away from long-range β^−^-emitters—such as ^32^P and ^89^Sr, which often suffered from severe hematological toxicity due to indiscriminate crossfire radiation—toward high-linear energy transfer (LET), short-range α-emitters (^223^Ra, ^225^Ac) and nanometer-scale Auger electron emitters. This physical constraint drastically spares healthy hematopoietic bone marrow while maximizing localized, irreparable double-strand DNA damage within malignant cell nuclei.

Second, the widespread adoption of flexible bifunctional chelation chemistry has decoupled the targeting vector from the radiometal core, giving rise to true “see-and-treat” theranostic pairs (such as ^68^Ga/^177^Lu, ^99m^Tc/^188^Re, and ^68^Ga/^225^Ac). By utilizing chemically identical or closely analogous diagnostic and therapeutic partners, clinicians can achieve exact pre-therapeutic patient stratification, perform precise quantitative image-based dosimetry, and monitor therapeutic response dynamically in real time.

Finally, a profound paradigm shift has occurred in the biological targets themselves. Historically, bone radiopharmaceuticals focused almost exclusively on the indirect, secondary host response—namely, osteoblastic mineral remodeling and hydroxyapatite chemisorption. Modern nuclear oncology increasingly bypasses this non-specific mineralized matrix to directly target biomarker-expressing tumor cells and their active cellular microenvironment, as dramatically demonstrated by the clinical success of PSMA- and FAPI-targeted theranostics, as well as novel genomic-level agents like PARP inhibitors.

### 11.4. The Artificial Intelligence Revolution: Digital Twins and Smart Dosimetry

Looking to the horizon, Artificial Intelligence (AI) and deep learning are poised to catalyze the next major transformation in skeletal radiotheranostics. Current clinical practice still largely relies on empirical, administered-activity dosing regimens. AI-driven image segmentation, automated radiomics, and deep-learning-based voxel dosimetry will enable patient-specific 3D Monte Carlo simulations in real time. By integrating multi-omic clinical datasets with imaging biomarkers, AI models will facilitate the creation of “digital twins”—virtual patient representations capable of predicting individual bone marrow toxicity thresholds, organ-at-risk absorbed doses, and therapeutic response prior to the first administration. Beyond image-based dosimetry, generative machine learning platforms are expanding directly into early-stage radiopharmaceutical discovery. These algorithms now enable in silico smart molecular design, predicting ligand–receptor binding affinities, radiometal–chelator stabilities, and whole-body pharmacokinetics prior to wet-lab synthesis, thereby vastly accelerating the translational pipeline for novel skeletal theranostic agents [172].

### 11.5. Combinatorial Paradigms and Future Frontiers

Despite remarkable breakthroughs, key challenges remain. The clinical management of recoiling radioactive daughters during α-decay cascades (e.g., ^225^Ac or ^223^Ra) demands improved nanocarrier containment or local retention strategies to prevent off-target renal and hematological toxicities. Moreover, the future of bone theranostics lies in multi-targeted combinatorial regimens. Synergizing targeted alpha or Auger therapy with immune checkpoint inhibitors, DNA damage response modifiers (such as PARP inhibitors), or bone-protective antiresorptive agents (denosumab) offers a realistic pathway to overcoming radiation resistance and preventing skeletal-related events.

Crucially, targeted radionuclide therapy provides a distinct systemic advantage over focal external beam radiotherapy by delivering immunomodulatory radiation across all disseminated skeletal niches simultaneously without precipitating severe systemic lymphopenia. At continuous low dose rates, this systemic radiation exposure transiently depletes exhausted tumor-infiltrating lymphocytes, upregulates cellular MHC-I and NK-activating ligands (e.g., NKG2D), and triggers immunogenic cell death. The resulting release of damage-associated molecular patterns (DAMPs) and tumor antigens establishes a broad antigenic cascade, priming systemic T-cell immunity against heterogeneous tumor clones across all metastatic sites to enhance checkpoint inhibitor efficacy [104].

To optimize the pharmacokinetics of small targeting vectors, strategic molecular engineering is essential. Conjugating low-molecular-weight reversible albumin binders extends systemic circulation kinetics, thereby aligning vector clearance with longer-lived therapeutic radionuclides to increase target site uptake and therapeutic efficacy [173]. Concurrently, pretargeting strategies utilizing bioorthogonal click reactions decouple the administration of the target-seeking vector from radionuclide delivery, offering exceptionally high target-to-background contrast and reduced whole-body radiation exposure when employing short-lived radiotracers [173].

Furthermore, the molecular targeting spectrum is expanding beyond classical pathways to novel surface antigens (including Nectin-4, uPAR, and integrin αv β6), which enable highly selective lesion detection with minimal non-target background accumulation [117]. This expansion is complemented by the introduction of innovative theranostic isotope pairs, such as ^212^Pb, ^161^Tb, and ^64^Cu/^67^Cu, which provide tailored physical emissions for matched diagnostic imaging and targeted endoradiotherapy [117].

Beyond static targeting vectors, the integration of stimulus-responsive release systems (such as pH-, redox-, and hyperthermia-sensitive nanocarriers) represents a promising frontier for skeletal radiotheranostics. By exploiting the acidic and ROS-rich microenvironment characteristic of active osteolytic lesions, these smart platforms enable trigger-specific payload release directly within the metastatic niche, enhancing localized radiation delivery while minimizing off-target bone marrow exposure [172]. Concurrently, radiolabeled nanotheranostic platforms (including liposomes, polymeric micelles, and inorganic nanoparticles) capitalize on the Enhanced Permeability and Retention (EPR) effect inherent to disrupted metastatic vasculature. When combined with active bone-seeking ligands, this passive accumulation strategy provides a dual-targeting mechanism that significantly elevates the therapeutic index compared to conventional small-molecule agents [172,174].

In conclusion, the evolution of bone radiopharmaceuticals from palliative, non-specific bone-seeking elements to sub-cellular, target-directed theranostic agents represents a triumph of interdisciplinary science. As nuclear chemistry, computational physics, and artificial intelligence continue to converge, bone-targeted radionuclide therapy stands on the threshold of substantially improving therapeutic efficacy and clinical outcomes in advanced metastatic disease.

## Figures and Tables

**Table 1:** Comprehensive Table of Diagnostic and Therapeutic Bone-Matrix-Targeting (Conventional) Radiopharmaceuticals.

Nuclide	Typical Ligand or Chemical Form (Development Status)	Primary Emission	Medical Application	Half-Life	Imaging Modality	LET Value ** (keV/µm)
^18^F	NaF (Sodium Fluoride)	β+	Diagnosis	109.77 min	PET	Low (<1)
^32^P	Sodium orthophosphate	β−	Therapy (Hist.)	14.26 d	(Bremss.)	Low (~0.2)
^45^Ca	Free cation (Ca^2+^) **[PC]**	β−	Therapy (Hist.)	162.6 d	-	Low (~0.2)
^47^Ca	Free cation (Ca^2+^) **[PC]**	β−	Diag./Ther.	4.54 d	(Bremss.)	Low (~0.2)
^68^Ga	Bisphosphonates (BPAMD **[C],** DOTAZOL **[C]**)	β+	Diagnosis	67.71 min	PET	Low (<1)
^89^Sr	Strontium Chloride (SrCl_2_/Metastron)	β−	Therapy	50.53 d	(Bremss.)	Low (~0.2)
^90^Y	Bisphosphonates (EDTMP **[C]**, DOTMP **[C]**)	β−	Therapy	64.1 h	(Bremss.)/PET	Low (~0.2)
^99m^Tc	MDP, HMDP, HDP	γ	Diagnosis	6.02 h	SPECT	Low (<1)
^109^Pd	Hydroxyapatite (HA) **[PC]**	β−	Therapy	13.7 h	Bremss./SPECT	Low (~0.2)
^117m^Sn	DTPA **[C]**	CE Auger	Therapy	13.6 d	SPECT	High (local)
^131^I	BP (Bisphosphonates) **[C]**	β−\γ	Diag./Ther.	8.02 d	SPECT	Low (~0.2)
^153^Sm	EDTMP (Lexidronam/Quadramet)	β−\γ	Therapy	46.5 h	SPECT	Low (~0.2)
^161^Tb	DOTAZOL **[PC]**	β−\γ, Auger	Therapy	6.89 d	SPECT	Low β/High Auger
^166^Ho	EDTMP **[C]**	β−\γ	Therapy	26.8 h	SPECT/MRI	Low (~0.2)
^177^Lu	EDTMP **[C]**, IBA **[C]** DOTAZOL **[C]**	β−\γ	Therapy	6.65 d	SPECT	Low (~0.2)
^186^Re	HEDP	β−\γ	Therapy	3.72 d	SPECT	Low (~0.2)
^188^Re	HEDP **[C]**, Ibandronate **[C]**, Pamidronate **[C]**	β−\γ	Therapy	17.0 h	SPECT	Low (~0.2)
^193m^Pt	BP (Bisphosphonates) **[PC]**	EC/CE/Auger	Diag./Ther.	4.33 d	SPECT	High (e)
^195m^Pt	BP (Bisphosphonates) **[PC]**	IT/CE/Auger	Diag./Ther.	4.02 d	SPECT	High (local)
^223^Ra	Radium chloride (RaCl_2_)	α\γ	Therapy	11.43 d	SPECT	High (80–100+)
^224^Ra	Radium chloride (RaCl_2_)	α	Therapy (Hist.)	3.63 d	N/A	High (80–100+)
^225^Ac	IBA **[PC]**, DOTAZOL **[PC]**	α	Therapy	10.0 d	SPECT (Daughter)	High (80–100+)
^227^Th	EDTMP **[PC]**, DTMP **[PC]**, DOTMP **[PC]**	α\γ	Therapy	18.68 d	SPECT	High (80–100+)

** LET (Linear Energy Transfer): Low LET: Most diagnostic isotopes (gamma and positron emitters) and traditional therapeutic beta emitters (^177^Lu, ^131^I, ^90^Y) deliver energy over a range of millimeters with low density, typically around 0.2 keV/µm. High LET: Alpha emitters (^223^Ra, ^225^Ac, ^211^At) have high LET values, generally ranging from 50 to 230 keV/µm. This results in dense ionization along a very short track (<100 µm), causing lethal double-strand DNA breaks. Auger and Conversion Electrons (CE): Isotopes like ^161^Tb and ^117m^Sn emit low-energy electrons that have high local LET at the nanometer or micrometer scale, delivering highly localized energy deposition at the nanometer scale.

**Table 2:** Comprehensive Table of Diagnostic and Therapeutic Direct Tumor- and Microenvironment-Targeting Bone-Related Radiopharmaceuticals.

Nuclide	Typical Ligand or Chemical Form (Development Status)	Primary Emission	Medical Application	Half-Life	Imaging Modality	LET Value ** (keV/µm)
^18^F	FDG, Choline, PSMA	β+	Diagnosis	109.77 min	PET	Low (<1)
^11^C	Acetate, Choline, L-Methionine **[C]**	β+	Diagnosis	20.39 min	PET	Low (<1)
^64^Cu	DOTATATE, PSMA ligands **[C]**	β+\β−	Diag. (Ther.)	12.7 h	PET	Low (<1)
^68^Ga	PSMA-11, DOTATATE	β+	Diagnosis	67.71 min	PET	Low (<1)
^89^Zr	Monoclonal Antibodies (Immuno-PET) **[C]**	β+	Diagnosis	78.41 h	PET	Low (<1)
^90^Y	Microspheres, DOTATATE **[C]**	β−	Therapy	64.1 h	(Bremss.)/PET	Low (~0.2)
^99m^Tc	DMSA, Sestamibi	γ	Diagnosis	6.02 h	SPECT	Low (<1)
^103^Pd	Brachytherapy seeds	EC/Auger	Therapy	16.99 d	X-ray/CT-	High (local)
^109^Pd	Porphyrins (Pd-TCPP) **[PC]**, Microspheres **[PC]**	β−	Therapy	13.7 h	Bremss./SPECT	Low (~0.2)
^111^In	Pentetreotide, Octreotide, DTPA, Antibodies **[C]**	γ	Diagnosis	2.8 d	SPECT	Low (<1)
^123^I	Sodium Iodide (NaI), MIBG, Ioflupane	γ	Diagnosis	13.27 h	SPECT	Low (<1)
^124^I	Sodium Iodide (NaI) **[C]**, MIBG **[C]**	β+	Diagnosis	4.18 d	PET	Low (<1)
^125^I	Seeds, Sodium Iodide (NaI), HSA, MIBG	EC/Auger	Diag./Ther.	59.4 d	SPECT/N/A	High (local)
^131^I	Sodium Iodide (NaI), MIBG	β−\γ	Diag./Ther.	8.02 d	SPECT	Low (~0.2)
^149^Tb	DOTANOC **[PC]**, Antibodies **[PC]**	α\β+\γ	Therapy	4.12 h	PET/SPECT	140–142
^161^Tb	DOTA-PSMA **[C]**, DOTATATE **[C]**	β−\γ, Auger	Therapy	6.89 d	SPECT	Low β/High Auger
^166^Ho	Microspheres (QuiremSpheres) **[C for bone]**	β−\γ	Therapy	26.8 h	SPECT/MRI	Low (~0.2)
^177^Lu	PSMA, DOTATATE	β−\γ	Therapy	6.65 d	SPECT	Low (~0.2)
^193m^Pt	Cisplatin **[PC]**	EC/CE/Auger	Diag./Ther.	4.33 d	SPECT	High (local)
^195m^Pt	Cisplatin **[PC]**	IT/CE/Auger	Diag./Ther.	4.02 d	SPECT	High (local)
^211^At	MABG **[C]**, FAPI **[C]**, Antibodies **[C]**	α	Therapy	7.21 h	SPECT/γ	99
^212^Pb	DOTAM-TATE **[C]**, PSMA **[C]**, Antibodies **[C]**	β−\γ	Therapy	10.64 h	SPECT	High (via daughter)
^213^Bi	DOTATOC, Antibodies, Substance-P	α\β−\γ	Therapy	45.59 min	SPECT	High (~100)
^224^Ra	DaRT seeds **[C]**	α	Therapy (Hist.)	3.63 d	N/A	High (80–100+)
^225^Ac	PSMA **[C]**, DOTATATE **[C]**	α	Therapy	10.0 d	SPECT (Daughter)	High (80–100+)

** LET (Linear Energy Transfer): Low LET: Most diagnostic isotopes (gamma and positron emitters) and traditional therapeutic beta emitters (^177^Lu, ^131^I, ^90^Y) deliver energy over a range of millimeters with low density, typically around 0.2 keV/µm. High LET: Alpha emitters (^223^Ra, ^225^Ac, ^211^At) have high LET values, generally ranging from 50 to 230 keV/µm. This results in dense ionization along a very short track (<100 µm), causing lethal double-strand DNA breaks. Auger and Conversion Electrons (CE): Isotopes like ^161^Tb and ^117m^Sn emit low-energy electrons that have high local LET at the nanometer or micrometer scale, delivering highly localized energy deposition at the nanometer scale.

## Data Availability

No new data were created or analyzed in this study. Data sharing is not applicable to this article.

## Abbreviations

The following abbreviations are used in this manuscript:

AE/AEs	Auger Electron/Auger Electrons
AI	Artificial Intelligence
ALP	Alkaline Phosphatase
BFC	Bifunctional Chelator
BP	Bisphosphonate
BPAMD	Bisphosphonate derivative/DOTA-bisphosphonate derivative
CE	Conversion Electron
CT	Computed Tomography
DOTA	1,4,7,10-tetraazacyclododecane-1,4,7,10-tetraacetic acid
DOTAM	1,4,7,10-tetrakis(carbamoylmethyl)-1,4,7,10-tetraazacyclododecane
DOTATATE DOTATOC DOTANOC	DOTA-conjugated somatostatin analogs
DOTAZOL	DOTA-conjugated zoledronate derivative
DOTMP	1,4,7,10-tetraazacyclododecane-1,4,7,10-tetrakis(methylenephosphonic acid)
DMSA	Dimercaptosuccinic acid
DTPA	Diethylene triamine pentaacetic acid
DTMP	Diethylenetriamine tetramethylene phosphonic acid
EC	Electron Capture
EDTMP	Ethylenediamine tetramethylenephosphonic acid
EGF	Epidermal Growth Factor
EGFR	Epidermal Growth Factor Receptor
EMA	European Medicines Agency
FAPI	Fibroblast Activation Protein Inhibitor
FDA	Food and Drug Administration
FDG	Fluorodeoxyglucose
FGFs	Fibroblast Growth Factors
HDP/HMDP	Hydroxymethylene diphosphonate
HEDP	Hydroxyethylidene diphosphonate
HEDTA	N-hydroxyethylene diamine triacetic acid
HYNIC	Hydrazinonicotinamide
IBA	Ibandronate
IGFs	Insulin-like Growth Factors
IL-6	Interleukin-6
LET	Linear Energy Transfer
MABG	Meta-alpha-astatobenzylguanidine
MAG3	Mercaptoacetyltriglycine
mCRPC	Metastatic Castration-Resistant Prostate Cancer
MDP	Methylene Diphosphonate
MIBG	Metaiodobenzylguanidine
MRI	Magnetic Resonance Imaging
NaF	Sodium Fluoride
NOTA	1,4,7-triazacyclononane-1,4,7-triacetic acid
NSCLC	Non-Small Cell Lung Cancer
OPG	Osteoprotegerin
PARP	Poly(ADP-ribose) Polymerase
PARPi	PARP Inhibitor
PET	Positron Emission Tomography
PSA	Prostate-Specific Antigen
PSMA	Prostate-Specific Membrane Antigen
PTHrP	Parathyroid Hormone-related Protein
RANK	Receptor Activator of Nuclear Factor-κB
RANKL	Receptor Activator of Nuclear Factor-κB Ligand
SPECT	Single Photon Emission Computed Tomography
SREs	Skeletal-Related Events
SUV	Standardized Uptake Value
TAT	Targeted Alpha Therapy
TGF-β	Transforming Growth Factor-beta
TME	Tumor Microenvironment
TNF-α	Tumor Necrosis Factor-alpha
TRT	Targeted Radionuclide Therapy
TTP	Time to Progression
VEGF	Vascular Endothelial Growth Factor
VEGFR	Vascular Endothelial Growth Factor Receptor
WBRT	Whole-Brain Radiation Therapy
WHO	World Health Organization
ZOL	Zoledronic Acid/Zoledronate
